# Structure and Dynamics in the ATG8 Family From Experimental to Computational Techniques

**DOI:** 10.3389/fcell.2020.00420

**Published:** 2020-06-10

**Authors:** Valentina Sora, Mukesh Kumar, Emiliano Maiani, Matteo Lambrughi, Matteo Tiberti, Elena Papaleo

**Affiliations:** ^1^Computational Biology Laboratory, Center for Autophagy, Recycling and Disease, Danish Cancer Society Research Center, Copenhagen, Denmark; ^2^Translational Disease System Biology, Faculty of Health and Medical Sciences, Novo Nordisk Foundation Center for Protein Research, University of Copenhagen, Copenhagen, Denmark

**Keywords:** molecular dynamics, structural biology, selective autophagy, short linear motifs, LIR motif

## Abstract

Autophagy is a conserved and essential intracellular mechanism for the removal of damaged components. Since autophagy deregulation is linked to different kinds of pathologies, it is fundamental to gain knowledge on the fine molecular and structural details related to the core proteins of the autophagy machinery. Among these, the family of human ATG8 proteins plays a central role in recruiting other proteins to the different membrane structures involved in the autophagic pathway. Several experimental structures are available for the members of the ATG8 family alone or in complex with their different biological partners, including disordered regions of proteins containing a short linear motif called LC3 interacting motif. Recently, the first structural details of the interaction of ATG8 proteins with biological membranes came into light. The availability of structural data for human ATG8 proteins has been paving the way for studies on their structure-function-dynamic relationship using biomolecular simulations. Experimental and computational structural biology can help to address several outstanding questions on the mechanism of human ATG8 proteins, including their specificity toward different interactors, their association with membranes, the heterogeneity of their conformational ensemble, and their regulation by post-translational modifications. We here summarize the main results collected so far and discuss the future perspectives within the field and the knowledge gaps. Our review can serve as a roadmap for future structural and dynamics studies of the ATG8 family members in health and disease.

## Introduction

Autophagy, a lysosomal self-eating process, is a conserved mechanism to maintain cellular homeostasis by recycling cellular components in response to nutrient shortage and by removing dysfunctional organelles and proteins in eukaryotic cells (Parzych and Klionsky, 2013; Bento et al., 2016; Mercer et al., 2018). During autophagy, an isolation membrane engulfs cargo by forming a double membrane vesicle called the autophagosome, which fuses with a lysosome where the material is degraded and recycled.

**FIGURE 1:**
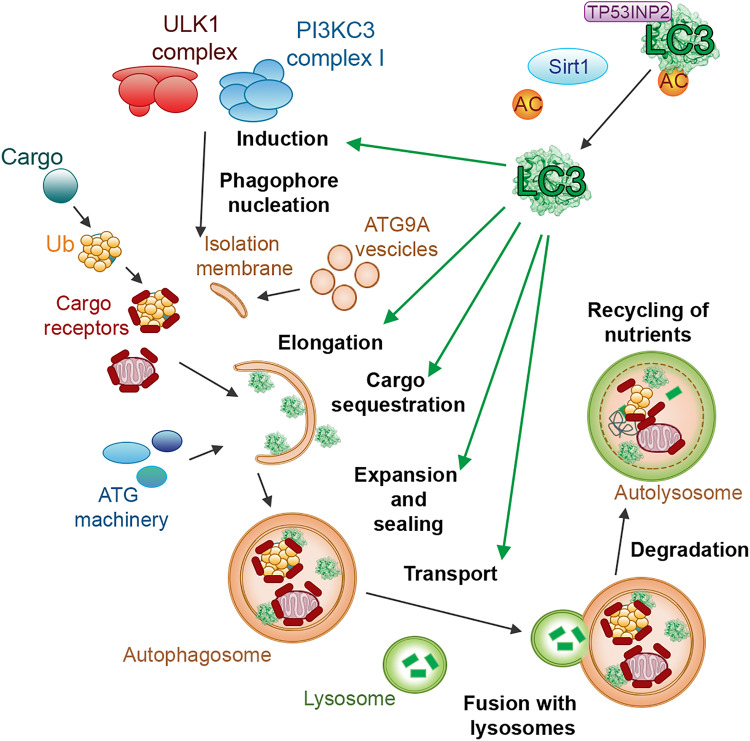
LC3 proteins in selective autophagy. The figure illustrates, as a reference for the reader, the involvement of the LC3 subfamily of ATG8 proteins in different steps of selective autophagy.

Thirty-six proteins are especially important for autophagy, out of which 16 belong to the core autophagy machinery (Suzuki et al., 2017). The autophagy proteins (ATGs) can be classified into six functional groups: (1) The ULK1–ATG13–RBCC1 (also called FIP200)–ATG101 complex; (2) the PtdIns3K class III complex containing VPS34, VPS15, and Beclin1; (3) the vesicles including the multi-spanning transmembrane protein ATG9; (4) the PtdIns3P-binding WIPI/ATG18–ATG2 complex; (5) the ubiquitin-like ATG5/ATG12 system and (6) the ubiquitin-like ATG8/LC3-PE conjugation system (Ohsumi, 1999; Suzuki et al., 2017). The last functional group (Figure 1) is essential in selective autophagy (Fimia et al., 2013; Zaffagnini and Martens, 2016; Gatica et al., 2018; Kirkin and Rogov, 2019). In selective autophagy, specific cargo (i.e., autophagy substrates) is selectively recruited by intracellular autophagy receptors and adaptors via ATG8 proteins and targeted into the autophagosome for subsequent degradation (Fimia et al., 2013; Zaffagnini and Martens, 2016; Gatica et al., 2018). ATG8 proteins have a central role in selective autophagy, as they recognize and bind different autophagy receptors and adaptors containing a specific class of short-linear motifs (SLiMs), see section “The Interaction Between Human ATG8 Proteins and Their Biological Partners Through Short Linear Motifs, i.e., the LC3 Interacting Regions (LIRs).” An emerging concept is that the human ATG8 proteins loaded with autophagy receptors bind to the concave surface of the phagophore membrane (inner membrane), whereas ATG8 proteins loaded with adaptors bind to the convex surface (outer membrane) of the autophagosome. As a consequence, autophagosomes recruit autophagy receptors together with the ubiquitinated cargo for degradation. On the contrary, autophagy adaptors are maintained intact (Johansen and Lamark, 2011; Stolz et al., 2014).

Despite the importance of ATG8 proteins in selective autophagy, several aspects of their mechanism of action are still unknown. Different members of the ATG8 family, such as the LC3 and GABARAP subgroups, can behave differently in various contexts and be specific for different LIR-containing interactors. For example, GABARAP, and not LC3 proteins, are involved in the activation of ULK1 (Joachim and Tooze, 2016). In contrast, the recruitment of the autophagy receptor p62 (also called SQSTM1) into the lysosome is dependent on the lipidation of LC3 proteins and does not involve GABARAP proteins (Shvets et al., 2011).

Autophagy modulation may also provide new means for the treatment of human pathologies, including cancer and neurodegeneration (Martinet et al., 2009; Gomes et al., 2016; Mancias and Kimmelman, 2016). Very little is known of the impact of disease-related alterations on the structure and function of the human ATG8 proteins. The availability of several experimental three-dimensional (3D) structures for ATG8 proteins and their interactors are opening new directions in the field of molecular modeling and simulations, which could become useful tools to integrate and complement the experimental research in autophagy. In this review, we aim at summarizing the most relevant results on the structure and interactions of human ATG8 proteins, along with discussing the first computational investigations of these proteins. In the first part of the review, we introduce the 3D structure and classification of ATG8 proteins. This is followed by a recap of the achievements in the field of experimental and computational structural biology on ATG8 proteins. In the second part of the review, we report the available structural studies on the biological interactions of ATG8 proteins, including binding to LIRs and other SLiMs, and biological membranes. Our final goal is to provide a comprehensive curation of structural data on human ATG8 proteins to guide future studies with molecular modeling and simulations. We also discuss outstanding questions in each of these fields of research.

## ATG8 Family Members in Human and Their Conservation

ATG8 proteins are highly conserved in eukaryotes (King, 2012) and exist in the form of one or several orthologs (Shpilka et al., 2011). Yeast has one single ATG8 protein, while higher organisms account for two or more ATG8 family members. Six mammalian ATG8 orthologs have been reported as central players of autophagy involved in protein transport, membrane remodeling, phagophore elongation, and closure (Antón et al., 2016), whereas two additional ones (GABARAPL3 and LC3B2) need additional experimental validation. Based on sequence similarity, the human ATG8 proteins can be classified into LC3 and GABARAP subfamilies. The LC3 subfamily includes LC3A, LC3B, and LC3C, whereas the GABARAP subfamily accounts for GABARAP, GABARAPL1, and GABARAPL2 (also called GATE-16). Two splicing variants of LC3A (i.e., LC3A-a and LC3A-b) have been reported at the same chromosomal position (20q11.22), displaying 98% of sequence similarity (Schaaf et al., 2016). LC3C and GABARAPL2 show little divergence in the phylogenetic analyses with respect to the subfamily to which they belong (Shpilka et al., 2011). LC3 proteins are involved in the initial steps of autophagosome formation and membrane expansion. GABARAP proteins mainly function at later stages of autophagosome formation, maturation and closure (Nguyen et al., 2016).

**FIGURE 2:**
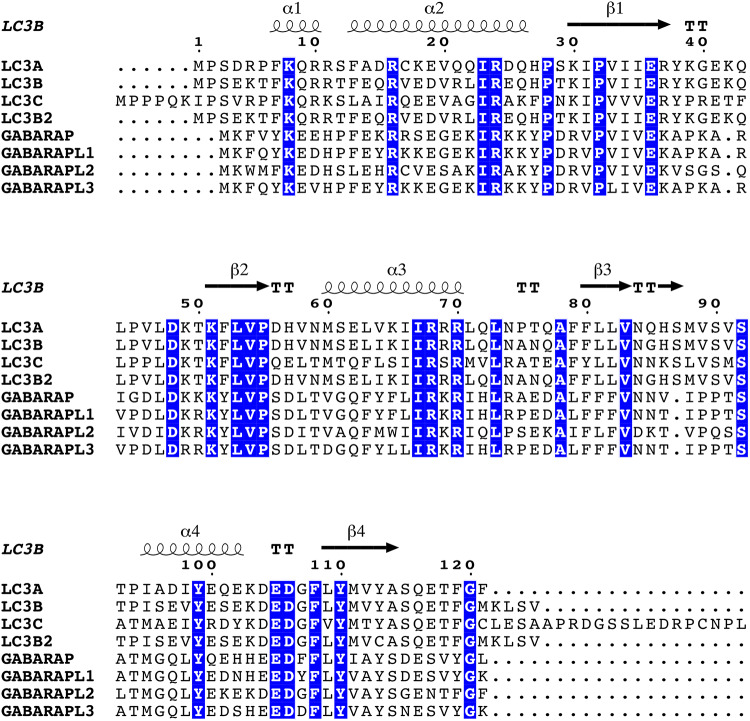
Multiple sequence alignment of human ATG8 family members. We used *Clustal Omega* (Madeira et al., 2019) to generate a multiple sequence alignment of all the human ATG8 proteins (LC3A, LC3B, LC3C, LC3B2, GABARAP, GABARAPL1, GABARAPL2, and GABARAPL3), after retrieving the corresponding FASTA sequences from *UniProt* (Bateman, 2019). The secondary structure definition has been calculated using DSSP (Kabsch and Sander, 1983; Joosten et al., 2011), and as a reference an experimentally resolved structure of LC3B [PDB ID 3VTU (Rogov et al., 2013)]. The figure was generated using the *ESPript* 3.0 web server (Robert and Gouet, 2014) with default parameters for the calculation of the conservation scores and tuning the graphical representation of the alignment. Positions featuring highly conserved residues are colored with a blue background.

Human ATG8 family members share from 29 to 94% sequence identity between them (Figure 2). In the LC3 subfamily, LC3B and LC3B2 are very similar to LC3A (∼ 80% of sequence identity), whereas LC3C is the one featuring higher diversity (∼ 50%). The GABARAP subfamily follows a similar pattern, with GABARAPL1 and GABARAPL3 close to GABARAP (∼ 80%), and GABARAPL2 with lower sequence identity (∼ 50%) (Schaaf et al., 2016). A recent computational study of ATG8 proteins from 20 different species revealed 68 gene duplication events, which eventually led to the differentiation and development of various subfamilies and their separation in higher eukaryotes at distinct chromosomal locations. The study also indicates that LC3A/B and GABARAP/GABARAPL1 could originate from the same phylogenetic node, whereas LC3C and GABARAPL2 branch into separate clades (Jatana et al., 2019). In the same study, the sequence-based analyses were accompanied by coevolution measurements and molecular modeling, suggesting that the GABARAP subfamily has a lower propensity to acquire alternative functions with respect to the LC3 subfamily (Jatana et al., 2019).

## Structure of ATG8 Family Members

**FIGURE 3:**
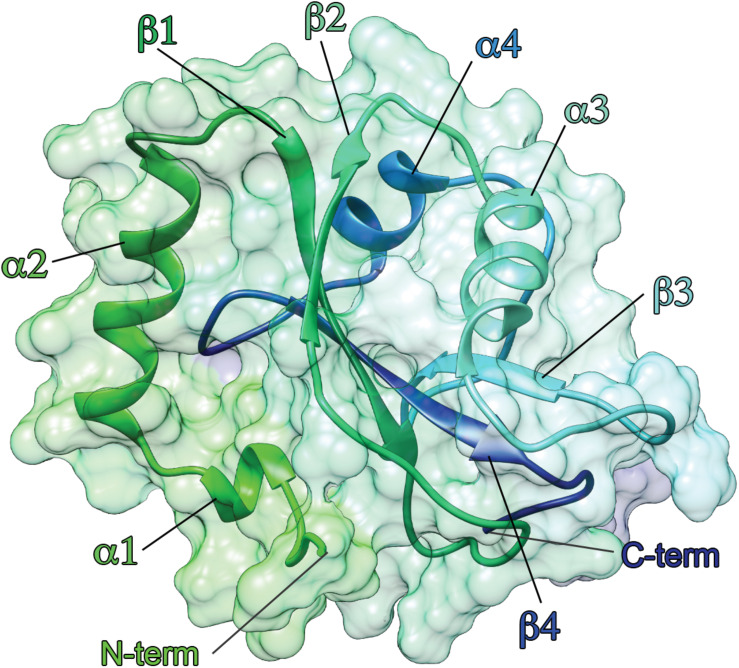
Three-dimensional architecture of ATG8 family members. The structure of LC3B is showed as an example. ATG8 proteins are ubiquitin-like protein, characterized by two α-helices at the N-terminal followed by a ubiquitin-like core. The structure of LC3B [PDB entry: 1V49 (Kouno et al., 2005)] is shown as cartoon and surface, using a color gradient from the N-terminal (green) to the C-terminal (dark blue).

Several experimental structures of ATG8 proteins have been deposited in the Protein Data Bank (PDB), mostly solved by X-ray crystallography and, in some cases, NMR spectroscopy (Supplementary Table S1). The 3D architecture is conserved among all members. ATG8 proteins are small proteins (14–16 kD) and contain a highly conserved ubiquitin-like core decorated by two extra N-terminal α-helices, i.e., α1 and α2, as shown in Figure 3 (Ichimura et al., 2008). There are differences in the N-terminal domain residues among the ATG8 members, which might be related to their preferences toward specific substrates (Jatana et al., 2019), as recently reported for LC3C (Krichel et al., 2019). The electrostatic potential surface of the N-terminal domain of the LC3 subfamily is highly basic, whereas that of the GABARAP subfamily is relatively acidic, except for GABARAPL1, which shows a neutral electrostatic potential surface (Sugawara et al., 2004). The ubiquitin-like core consists of four beta strands (β1, β2, β3, and β4) surrounding two helices, i.e., α3 between the β2 and β3 strands and α4 between the β3 and β4 strands (Shpilka et al., 2011).

**FIGURE 4:**
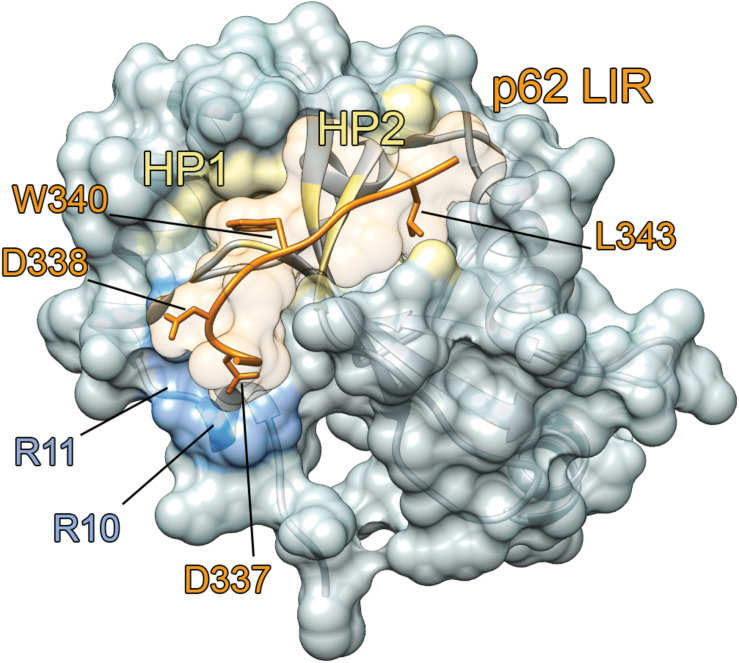
LIR-mediated interaction with ATG8 proteins. The complex between LC3B and p62 [PDB entry: 2ZJD (Ichimura et al., 2008)] is reported as an example. LC3B mediates protein-protein interactions and recruits the autophagy receptors through the binding of a short linear motif, called LIR. The complex of LC3B (gray cartoon and surface) with the LIR motif of mammalian p62 (orange cartoon) is shown. The key residues for the binding of p62 LIR motif to LC3B are indicated as sticks. The two hydrophobic pockets (HP1 and HP2) and the R10 and R11 in the binding interface of LC3B are indicated in yellow and light blue, respectively.

One of the most important features of ATG8 proteins is the presence of two hydrophobic grooves in their N-terminal domain (Ichimura et al., 2008), named hydrophobic pockets HP1 and HP2 (Figure 4). The two hydrophobic pockets are quite conserved among the ATG8 proteins, and they can accommodate conserved residues of autophagy adaptors and receptors, as detailed in section “The Interaction Between Human ATG8 Proteins and Their Biological Partners Through Short Linear Motifs, i.e., the LC3 Interacting Regions (LIRs).”

The HP1 pocket is generally more conserved than the HP2 one among the ATG8 family members (Noda et al., 2010). As an example of its composition, the HP1 pocket includes the side chains of D19, I23, P32, I34, K51, L53, and F108 in LC3B. In contrast, the HP2 pocket of LC3B includes the hydrophobic side chains of F52, V54, P55, L63, I66, and I67 (Noda et al., 2008; Kuang et al., 2016). These residues provide the platform for hydrophobic, electrostatic and hydrogen bond interactions with LIR-containing proteins (Birgisdottir et al., 2013; Fracchiolla et al., 2017; Wirth et al., 2019). Besides the two hydrophobic pockets, there are other two important conserved regions on the ATG8 structure, called the LDK tripeptide (i.e., L47, D48, and K49) and the ubiquitin patch (L8-I44-V70), which are located in the front and back of the HP1 and HP2 pocket, respectively. These patches can contribute to a variety of LIR-mediated interactions (Birgisdottir et al., 2013; Atkinson et al., 2019).

## Structural Dynamics of ATG8 Family Members

The ATG8 family members are among the autophagy proteins that have been investigated in more structural details (Weiergräber et al., 2017). In this context, the integration of experimental biophysical techniques with computational approaches based on molecular dynamics (MD) is promising, thanks to the complementary information that they provide (Esteban-Martín et al., 2012; Papaleo, 2015; Papaleo et al., 2016). Among different biophysical approaches, NMR spectroscopy has the unique capability of assessing protein dynamics over a wide range of timescales. Protein NMR allows collecting different parameters for proteins of the size of the ATG8 proteins, which account for local and long-range conformational changes (Palmer, 2004; Mittermaier and Kay, 2009; Tzeng and Kalodimos, 2011; Manley and Loria, 2012; Torchia, 2015). The integration of these experimental measurements with an atom-level description, like the one provided by MD simulations (Klepeis et al., 2009; Dror et al., 2012), can shed light on the different conformational states of ATG8 proteins and how they changes upon interaction with biological partners.

Biomolecular simulations still suffer from approximations. These approximations are mainly associated with the quality of the physical models used to describe the system (i.e., the force fields), and with the coverage of the conformational space accessible during the simulation (i.e., the sampling) (Klepeis et al., 2009; Lindorff-Larsen et al., 2012; Mobley, 2012). NMR-derived data can be used to evaluate the quality of an ensemble of protein structures collected by MD simulations and its agreement with the corresponding experimental data in solution. As an example, different algorithms are available to back-calculate NMR parameters such as backbone and side-chain chemical shifts from a structural ensemble (Li and Brüschweiler, 2012), or to estimate secondary structure content on a per-residue basis (Camilloni et al., 2012a). Therefore, it is possible to estimate, for example, the relative populations of secondary structures for each residue of the protein in solution and to compare them to secondary structures calculated from dictionaries, such as DSSP (Kabsch and Sander, 1983), or based on structural alphabets (Craveur et al., 2015). An example is our recent study on the benchmark of ten different MD force fields to study the conformational ensemble of LC3B (Aykac Fas et al., 2019). This first study guides force-field selection for future studies of ATG8 proteins with biomolecular simulations, along with providing a protocol to follow to evaluate other force fields. Several force fields provide a reasonable structural ensemble of LC3B. Our study also points out local differences, indicating that, depending on the selected physical models for protein and solvent, certain regions of the protein cannot be described accurately during the simulations. Nevertheless, the CHARMM22^∗^ force field (Piana et al., 2011) could be recommended to study LC3B according to our comparison.

The inherent issues with the sampling achieved by classical MD simulations can be overcome using enhanced sampling approaches (Bernardi et al., 2015; Spiwok et al., 2015). The availability of NMR parameters can come handy since they can be used in the simulation protocol, as in the case of NMR-derived replica-averaged restraints (Lindorff-Larsen et al., 2005; Camilloni et al., 2012b; Bonomi et al., 2017; Papaleo et al., 2018). As an example, the yeast ATG8 protein has been studied by all-atom MD simulations with methyl chemical shifts as replica-averaged restraints (Kannan et al., 2014), using the AMBER ff99SB^∗^-ILDN-Q force field (Best and Hummer, 2009; Lindorff-Larsen et al., 2010; Best et al., 2012). Here, the authors incorporated the experimental methyl resonances into the force field as a restraint potential. Other approaches are available to use backbone chemical shifts as restraints in simulations and are continuously improved in their formulation (Camilloni et al., 2012b; Löhr et al., 2017, 2019).

It should be noted that the major biological activities of ATG8 proteins depend on their membrane-bound state, as detailed in section “Interaction of ATG8 Family Members With Autophagic Membranes.” The dramatic decrease in solubility of lipid-conjugated ATG8 proteins challenges experimental techniques, so the majority of the biophysical studies in solution has been carried out on the non-lipidated forms (Stangler et al., 2002; Kouno et al., 2005; Kumeta et al., 2010; Schwarten et al., 2010; Klionsky et al., 2012; Rogov et al., 2013; Ma et al., 2015; Krichel et al., 2019). This is also true for the studies performed *in silico* with modeling and simulations (Ma et al., 2015; Di Rita et al., 2018; Holdgaard et al., 2019; Jatana et al., 2019). For such computational studies, the major challenge is the availability of good parameters to describe the lipid-conjugated form of the protein, an area that deserves future attention. Despite these difficulties, several studies shed light on the dynamics of lipidated forms of ATG8 by experimental approaches (see section “Interaction of ATG8 Family Members With Autophagic Membranes” for more details).

The structural studies of ATG8 proteins suggested that their conformational propensity and structural flexibility are important for their cellular functions and specificity (Weiergräber et al., 2017). A recent MD study using the OPLS force field highlighted differences in the pattern of intramolecular contacts in the proximity of the HP2 pocket, suggesting a role for this area in the modulation of the LIR recognition in different ATG8 proteins (Jatana et al., 2019). Our MD simulations of PCM1 LIR in complex with different human ATG8 proteins (Holdgaard et al., 2019) corroborate the idea that important differences in the LC3 and GABARAP subfamilies are related to different patterns of electrostatic interactions and hydrogen bonds. These differences are due to both the N-terminal and C-terminal regions flanking the core LIR motif and dictate diverse conformational propensities to accommodate the LIR in the ATG8 hydrophobic groove (Holdgaard et al., 2019; Wirth et al., 2019), as more extensively discussed in section “Specificity of Different ATG8 Family Members in LIR Recognition.” NMR relaxation analysis and NOE measurements highlighted ^15^N relaxation dispersions and line broadening of resonances in the N-terminal region of GABARAP, GABARAPL2, and LC3C. These data indicate a disordered N-terminal region and the presence of slow conformational exchange, involving the helices α1 and α2, and the loop α4-β4 (Stangler et al., 2002; Krichel et al., 2019). X-ray crystallography also suggested the existence of alternative conformations in the N-terminal region, as shown for the GABARAP subfamily (Coyle et al., 2002; Ma et al., 2015). Here, the changes are likely to be associated with rearrangements in the α1–α2 loop in the proximity of proline P10 of GABARAP (Coyle et al., 2002). Moreover, the electron density map for the N-terminal region of ATG8 proteins is often of difficult interpretation and associated with high crystallographic B factors, supporting the notion of a heterogeneous ensemble of conformations with a certain degree of disorder (Coyle et al., 2002). An enhanced N-terminal dynamics seems to be a characteristic of the yeast ATG8 variant, and can be reduced by mutating P26 to the corresponding lysine of the human orthologs (Kumeta et al., 2010). Furthermore, a recently deposited NMR structure of LC3C (Krichel et al., 2019) shows that its short α1 helix is not stable and consists of a polyproline II motif tethered to the rest of the protein core by a flexible linker. The role of the conformational heterogeneity in the N-terminal regions of ATG8 proteins is not fully understood. Different hypotheses have been formulated, such as a role in autophagosome formation, membrane tethering/fusion (Nakatogawa et al., 2007; Weidberg et al., 2011), recognition of the mitochondrial membrane (Chu et al., 2013), and interaction with the microtubule cytoskeleton (Krichel et al., 2019). We speculate that prolines at critical positions in the ATG8 structures could act as conformational switches (Andreotti, 2003). In this context, enhanced sampling MD approaches can support, for example, the investigation of cis-trans proline isomerization (Leone et al., 2009; Camilloni et al., 2014).

The C-terminal region of ATG8 proteins (i.e., the tract after the β4 strand) is also highly flexible and with a propensity to disorder, as indicated by NMR relaxation analysis and NOE measurements (Krichel et al., 2019). NMR relaxation measurements of GATE-16 also support the notion of a disordered C-terminal tract (residue 112–117), which populates different conformations in solution, from extended and solvent-accessible “open” states to “closed” conformations, forming interactions with the LIR binding surface (Ma et al., 2015). To gain atom-level details on these conformational changes, the authors used an enhanced sampling approach based on a combination of Hamiltonian Replica-Exchange and conventional MD using the AMBER ff99SB-ILDN (Lindorff-Larsen et al., 2010) and CHARMM27 (Bjelkmar et al., 2010) force fields. The combination of NMR and MD allowed the authors to identify several conformational states in dynamic equilibrium for the C-terminal region (Ma et al., 2015). The simulation data suggest a ‘swing-out’ movement depending on the F115 anchoring residue (Ma et al., 2015). The authors also suggest that an extended conformation of the C-terminal region can be selected for its proteolytic cleavage by ATG4 and favor the step of lipid conjugation at the terminal glycine residue (G120).

Another recent study highlights the importance of a solvent accessible C-terminal region for the formation of the ATG8-II forms (Zhao et al., 2017). The study focused on the molecular mechanisms associated with autophagy stimulation by epigallocatechin gallate (EGCG), a bioactive component of green tea with anticancerogenic potential (Zhao et al., 2017). The authors used all-atom MD simulations with the GROMOS96 53a6 force field (Oostenbrink et al., 2004) and suggested that EGCG could interact with the so-called LC3-I form, inhibit its dimerization and expose the C-terminal G120, promoting the formation of the LC3-II form.

Moreover, NMR and fluorescence resonance energy transfer (FRET) experiments have been applied to investigate the binding affinities and specificity between ATG8 proteins and different LIR-containing peptides, highlighting the role of interactions that are distal from the ATG8 hydrophobic pocket (Atkinson et al., 2019).

We foresee that the continuous developments in experimental and computational structural biology techniques, and especially in their integration, will allow a deeper understanding of the structure-function-dynamics relationship of ATG8 proteins and clarify the determinants of their specificity toward certain binding partners. These studies will be also crucial to investigate the roles of ATG8 proteins in disease and their potential as drug targets. In this context, we started a series of studies in which we have been linking data from cancer genomic initiatives with structural ensembles to understand the impact of cancer-related alterations in autophagy proteins, such as ULK1 (Kumar and Papaleo, 2019) and LC3 proteins (Aykac Fas et al., 2019). Our framework allows to predict different layers of changes that a mutation induces on the protein product, including alteration of its structural stability, post-translational modifications, the capability to interact with biological partners, both disclosing local effects at the binding interface and more elusive and allosterically induced distal effects. These structure-based annotations can be used to predict driver and passenger mutations, or to prioritize variants for experimental validation, selecting the proper biological readouts depending on the major effect that a mutation is predicted to elicit.

Advances in the simulation field and in the integration with NMR data could become an asset to elucidate molecular mechanisms associated with the dynamics of ATG8 proteins in the millisecond time scale. Moreover, simulations will allow studying more in detail the complexes between ATG8 proteins and their binding partners, including other proteins and biological membranes. On the other hand, advances in other experimental techniques, such as cryo-electron microscopy (Danev et al., 2019) and fluorescence spectroscopies (König et al., 2015), could open new directions for the study of large macromolecular assemblies which include ATG8 proteins, another field suitable for the integration of experimental biophysical measurements and MD simulations (Bonomi and Vendruscolo, 2019; Igaev et al., 2019).

Due to the potential of the integration of NMR and MD simulations for the study of ATG8 proteins, it would be beneficial to direct more efforts toward the collection of complete sets of NMR data, including full assignment of side-chain chemical shifts for methyl-containing residues of all the human ATG8 proteins in their free, membrane-associated, and LIR-bound states, along with NMR measurements such as long-range NOEs or Residual Dipolar Couplings. To facilitate a culture of responsible and effective data sharing, NMR data and MD trajectories of ATG8 proteins should be also stored in dedicated repositories (PLUMED Consortium, 2019), so that they can be used for re-analyses, contributing to boost the structural studies of this important class of autophagic proteins. To move a first step toward this goal, we have been providing the simulation data associated with our publications on ATG8 proteins. To mention an example, a GitHub repository^1^ contains the data of our molecular modeling and simulations studies of the PCM1 LIR (Holdgaard et al., 2019).

## The Interaction Between Human ATG8 Proteins and Their Biological Partners Through Short Linear Motifs, i.e., the LC3 Interacting Regions (LIRs)

As mentioned above, the LIR motif is the portion of the sequence of autophagy receptors and adaptor proteins that facilitates the selective recruitment of autophagy substrates to the autophagosome (Noda et al., 2010). LIR motifs can be classified as SLiMs, which are contiguous sequence tracts of disordered proteins characterized by degenerated sequences where a small number of highly conserved residues are located between more loosely conserved positions. They are essential for protein binding specificity and often cooperate with other SLiMs to increase the binding affinity to a partner of interaction (Davey et al., 2012).

LIR motifs, which are usually 15–20 amino acids long, are located in intrinsically disordered regions with a propensity to undergo disorder-to-order transitions (Popelka and Klionsky, 2015). Indeed, it has been reported that the LIR motif, in its ATG8-bound state, contributes to the formation of an extended intermolecular parallel β-sheet (Birgisdottir et al., 2013). The disordered character of LIR motifs might explain their structural and functional diversities (Popelka and Klionsky, 2015). Moreover, it could explain the micromolar to sub-micromolar binding affinity of LIRs to ATG8 proteins (Popelka and Klionsky, 2015).

We curated the known complexes between ATG8 proteins and LIR peptides from different interactors solved by X-ray crystallography or NMR (Supplementary Table S1) for a total of 46 3D structures of 38 complexes. Some of these structures include the phosphorylated or phosphomimetic variants of the LIR, as in the case of optineurin and PI3K type 3.

We noticed that, in most cases, the bound LIR peptide is in an extended conformation, with few exceptions where the C-terminal part of the extended LIR forms a helical structure. This is the case for FYCO1 (Cheng X. et al., 2016; Sakurai et al., 2017), RETREG1, Ankyrin-2, and Ankyrin-3 (Li et al., 2018). In the GABARAP-RETREG1 complex, the helical C-terminal region is predicted and remains to be validated (Li et al., 2018). Tandem LIR repeats can also occur (Kwon et al., 2017). The possibility of bent conformations has been suggested by our models and simulations of the complex between PCM1 and GABARAP (Holdgaard et al., 2019) and remains to be validated. LC3B can also bind a coiled-coil part of the retroviral restriction factor Trim5α (Keown et al., 2018); see section “The Core LIR Motif” for more details.

### The Core LIR Motif

LIRs have been reported with a four-residues core central sequence that, together with its adjacent residues, determines the binding to the ATG8 proteins. LIRs have a highly conserved unique core sequence represented as Φ_1_X_2_X_3_Ψ_4_, (where Φ_1_ = W/F/Y, Ψ_4_ = L/I/V, and X = any amino acid) responsible for the binding to the HP1 and HP2 hydrophobic pockets (Figure 4) of ATG8 proteins. In the majority of LIRs, the positions preceding the core sequence Φ_1_X_2_X_3_Ψ_4_ are usually occupied by acidic or phosphorylatable amino acids (see section “Post-Translational Modulation of the LIR-ATG8s Interaction”).

LIR whose core fits the canonical definition can be classified based on the aromatic amino acid in position Φ_1_ into W-type, Y-type, and F-type LIRs (Birgisdottir et al., 2013; Wild et al., 2014; Johansen and Lamark, 2019). W-type LIRs have a stronger binding to the HP1 pocket than F- and Y-type LIRs, likely due to steric effects (Birgisdottir et al., 2013). The higher binding affinity yielded by a tryptophan residue at the Φ_1_ position has been confirmed by experimental mutagenesis (Rozenknop et al., 2011; Rogov et al., 2013). The authors replaced the Φ_1_ residue of F- or Y-type LIRs with tryptophan and measured the associated dissociation constants (K_d_) with isothermal titration calorimetry and NMR chemical shift perturbation (Rozenknop et al., 2011; Rogov et al., 2013). We suggest that another determinant of the different binding affinity to the HP1 pocket could reside in optimized aromatic or amino-aromatic interactions when tryptophan is in Φ_1_ position, involving for example residues K51 and F108 of LC3B. This hypothesis could be explored using MD simulations together with polarizable force fields to better account for the nature of this interactions (Lemkul and MacKerell, 2017). The Y-type LIR motifs may play a unique role in cargo recognition and recruitment because the tyrosine may undergo phosphorylation or redox modifications during oxidative stress, which can modulate the amplitude of autophagy (Adams et al., 2015).

Another class of LIRs is the one of the non-canonical LIRs, which do not fit the sequence requirements described so far and are discussed in detail in section “Non-canonical LIR Motifs (CLIRs).”

Among the available complexes with known structures (Supplementary Table S1), the majority features a canonical core LIR motif, with two exceptions (see below). A recent study, which analyzed over 100 LIR sequences, shows how W and F are the most common residues in position Φ_1_, followed by Y (Johansen and Lamark, 2019). The complexes with known structures reflect this distribution, with 11 out of 23 canonical LIRs featuring tryptophan, ten a phenylalanine and two a tyrosine at position Φ_1_. Similarly, in our dataset, the residue occupying the Ψ_4_ position is an isoleucine (eight occurrences), a valine (seven), a leucine (seven) or a phenylalanine. Both Φ_1_ and Ψ_4_ residues are deeply buried into the HP1 and HP2 pockets. The core motif residues can also engage in hydrogen bonds between the backbone of the LIR peptide and the ATG8 protein. Nonetheless, in at least a few cases, subtle variations in the main binding mode have been observed, despite the presence of a canonical LIR sequence. This happens in the GABARAP-KBTBD6 complex, in which W668 occupies HP1, but the Ψ_4_ residue (V671) is in contact with the rim of HP2 instead of being buried (Genau et al., 2015). Moreover, R670 (X_3_ position) in the core motif of KBTBD6 interacts with a tyrosine (Y25) of GABARAP through a hydrogen bond. Y25 can also interact with a lysine in position X_3_ in the complex between GABARAP and the PCM1 LIR (Holdgaard et al., 2019; Wirth et al., 2019).

In the LC3B-FUNDC1 complex, Y18 (Φ_1_) and L21 (Ψ_4_) bind the HP1 and HP2 LC3B pockets as expected. However, V20 (X_3_) binds inside HP1, making hydrophobic contacts with the side chain of L53 of LC3B. This causes the E19 side chain (X_2_) to point away from the binding pocket and Y18 to be less buried into HP1 than in other ATG8s-LIR complexes. The importance of this interaction is supported by the fact that a V20A substitution in FUNDC1 significantly impairs its binding to LC3B (Kuang et al., 2016). Phosphorylation of FUNDC1 can rescue a canonical binding mode (Kuang et al., 2016). A mechanism based on phosphorylation to revert a LIR non-canonical binding mode to a canonical one could be a general mechanism for other LIR sequences and deserves further investigation.

As mentioned above, another variation is the binding mode of Trim5α to LC3B, where the LIR region is in a helical conformation. The Trim5α helical LIR occupies the position of the conventional LIRs and the residue Φ_1_ (W196) of the LIR binds to HP1, whereas HP2 remains unoccupied (Keown et al., 2018).

The X_2_ and X_3_ positions of LIR motifs are less conserved and often occupied by hydrophobic, acidic, or even basic amino acids (Johansen and Lamark, 2019). The sequences reported in Table 1 suggest that even more variability is allowed, with cases of polar residues such as threonine in X_2._ While X_2_X_3_ residues typically interact with the ATG8 binding site through their backbone, their side chains could occasionally be involved and account for specificity (see section “Specificity of Different ATG8 Family Members in LIR Recognition”).

**TABLE 1:** List of known interactors of ATG8 proteins for which a LIR-dependent binding has been experimentally validated by mutagenesis.

**Protein (UniProt recommended name, short name if available)**	**UniProt ID**	**LIR type**	**LIR core sequence**	**References (PMID)**
Activating molecule in BECN1-regulated autophagy protein 1 (AMBRA1)	Q9C0C7	W-type	1049-WDQL-1052	25215947 30217973
Ankyrin-2	Q01484	W-type	1592-WVIV-1595	29867141
Ankyrin-3	Q12955	W-type	1989-WIEF-1992	29867141
AP-2 complex subunit alpha-1 (AP2A1)	O95782	W-type	879-WKQL-882	24067654
Ataxin-3	P54252	F-type W-type	74-FFSI-77 130-WFNL-133	31625269
Atlastin-3	Q6DD88	F-type	390-FKQL-393	30773365
Autophagy-related protein 2A (ATG2A)	Q2TAZ0	F-type	1362-FCIL-1365	32009292
Autophagy-related protein 2B (ATG2B)	Q96BY7	F-type	1491-FCIL-1494	32009292
Autophagy-related protein 13 (ATG13)	O75143	F-type	444-FVMI-447	23043107
Bcl-2-like protein 13 (Bcl2-L-13)	Q9BXK5	W-type	276-WQQI-279	26146385
BCL2/adenovirus E1B 19 kDa protein-interacting protein 3 (BNIP3)	Q12983	W-type	83-WVEL-86	23209295 22505714
BCL2/adenovirus E1B 19 kDa protein-interacting protein 3-like (BNIP3L)	O60238	W-type	36-WVEL-39	20010802 28442745
Beclin 1-associated autophagy-related key regulator (Barkor/ATG14)	Q6ZNE5	W-type	435-WENL-438	30767700
Beclin1	Q14457	F-type	97-FTLI-100	30767700
C-Jun-amino-terminal kinase-interacting protein 1 (JIP-1)	Q9UQF2	F-type	336-FDCL-339	24914561
Cadherin-6	P55285	Y-type	764-YDYL-767	27375021
Calcium-binding and coiled-coil domain-containing protein 2 (CALCOCO2)	Q13137	non-canonical	134-LVV-136	23022382
Calreticulin	P27797	W-type	200-WDFL-203	30429217
Catenin beta-1	P35222	W-type	504-WPLI-507	23736261
Cell cycle progression protein 1 (CCPG1)	Q9ULG6	W-type	14-WTVI-17	29290589 31006538
Cryptochrome-1 (CRY1)	Q16526	Y-type	273-YKKV-276 287-YGQL-290 488-YQQL-491 494-YRGL-497	29937374
Cysteine protease ATG4A (ATG4A)	Q8WYN0	F-type	393-FEIL-396	28287329
Cysteine protease ATG4B (ATG4B)	Q9Y4P1	F-type	388-FEIL-391	28287329
Cysteine protease ATG4C (ATG4C)	Q96DT6	F-type	455-FVLL-458	28287329
Disrupted in schizophrenia 1 protein (DISC1)	Q9NRI5	F-type	210-FSFI-213	30488644
E3 ubiquitin-protein ligase NEDD4 (NEDD4)	P46934	W-type	685-WEII-688	28470758
Fas-apoptotic inhibitory molecule 2 (FAIM2)	Q9BWQ8	W-type	65-WAYV-68	31914609
FUN14 domain-containing protein 1 (FUNDC1)	Q8IVP5	Y-type	18-YEVL-21	22267086
FYVE and coiled-coil domain-containing protein 1 (FYCO1)	Q9BQS8	F-type	1280-FDII-1283	20100911 23043107
Golgi reassembly stacking protein 2 (GRS2)	Q9H8Y8	Y-type	196-YGYL-199	29297744
Hepatocyte growth factor receptor (HGF receptor)	P08581	Y-type	1234-YYSV-1237	30786811
Histone acetyltransferase KAT2A (KAT2A)	Q92830	Y-type	734-YTTL-737	31878840
Huntingtin	P42858	W-type	3035-WVML-338	25385587
Inhibitor of nuclear factor kappa-B kinase subunit alpha (IKK-A)	O15111	W-type W-type	651-WHLL-654 740-WSWL-743	29717061
Junction-mediating and -regulatory protein (JMY)	Q8N9B5	W-type	13-WVAV-16	26223951
Kelch repeat and BTB domain-containing protein 6 (KBTBD6)	Q86V97	W-type	668-WVRV-671	25684205
Kelch repeat and BTB domain-containing protein 7 (KBTBD7)	Q8WVZ9	W-type	668-WVQV-671	25684205
Mitochondrial antiviral-signaling protein (MAVS)	Q7Z434	Y-type	9-YKYI-12	27551434 28141795
Mitochondrial ubiquitin ligase activator of NFKB 1 (MUL1)	Q969V5	Y-type	327-YRAL-330	25224329
Mitogen-activated protein kinase 15 (MAPK15)	Q8TD08	Y-type	340-YQMI-343	22948227
Next to BRCA1 gene 1 protein (NBR1)	Q14596	Y-type	732-YIII-735	21620860
NLR family member X1 (NLRX1)	Q86UT6	F-type	463-FQLL-466	30804553
Nuclear fragile X mental retardation-interacting protein 1 (NUFIP1)	Q9UHK0	W-type	40-WAML-43	29700228
Nuclear receptor corepressor 1 (N-CoR1)	O75376	F-type	346-FPEI-349	30952864
Optineurin	Q96CV9	F-type	178-FVEI-181	25294927 23805866
Paxillin	P49023	Y-type	40-YQEI-43	27184837
Peptidyl-prolyl cis-trans isomerase FKBP8 (PPIase FKBP8)	Q14318	F-type W-type	24-FEVL-27 93-WLDI-96	28381481 31908024
Pericentriolar material 1 protein (PCM-1/PCM1)	Q15154	F-type	1963-FVKV-1966	31053714
Phosphatidylethanolamine-binding protein 1 (PEBP-1)	P30086	W-type	55-WDGL-58	27540684
Phosphatidylinositol 3-kinase catalytic subunit type 3 (PI3K type 3)	Q8NEB9	F-type	250-FELV-253	30767700
Pleckstrin homology domain-containing family M member 1 (PLEKHM1)	Q9Y4G2	W-type	635-WVNV-638	25498145 28655748
Prohibitin-2 (PHB2)	Q99623	Y-type	121-YQRL-124	28017329
Protein kinase C zeta type (PRKCZ)	Q05513	F-type	578-FEYI-581	31857374
Protein PML (PML)	P29590	Y-type F-type	119-YRQI-122 612-FFDL-615	25419843
RB1-inducible coiled-coil protein 1 (RBCC1)	Q8TDY2	F-type	702-FETI-705	23043107
Receptor of activated protein C kinase 1 (RACK1)	P63244	W-type W-type	132-WNTL-125 170-WDKL-173	27129200
Reticulon-3 (RTN3)	O95197	F-type Y-type F-type W-type F-type Y-type	205-FTLL-208 217-YSKV-220 248-FEVI-251 342-WDLV-345 555-FEEL-558 790-YDIL-793	28617241
Reticulophagy regulator 1 (RETREG1)	Q9H6L5	F-type	455-FELL-458	26040720
Segment polarity protein disheveled homolog DVL-2 (Disheveled-2/Dvl2)	O14641	W-type	444-WLKI-447	20639871
Sequestosome-1 (SQSTM1)	Q13501	W-type	338-WTHL-341	17580304 18653543 19812211 27158844
Serine/threonine-protein kinase 3 (STK3)	Q13188	non-canonical	365-MVI-367	31857374
Serine/threonine-protein kinase Nek9 (NEK9)	Q8TD19	W-type	967-WCLL-970	31857374
Serine/threonine-protein kinase ULK1 (ULK1)	O75385	F-type	357-FVMV-360	23043107
Serine/threonine-protein kinase ULK2 (ULK2)	Q8IYT8	F-type	353-FVLV-356	23043107
SHC-transforming protein 1 (SHC1)	P29353	Y-type	10-YNPL-13	30109811
Sprouty-related, EVH1 domain-containing protein 2 (Spred-2)	Q7Z698	W-type Y-type	378-WMAL-381 394-YLPL-397	27028858
Starch-binding domain-containing protein 1 (STBD1)	O95210	W-type	203-WEMV-206	21893048
Stimulator of interferon genes protein (hSTING)	Q86WV6	Y-type Y-type Y-type	167-YLRL-170 199-YILL-202 245-YELL-248	30568238
Tax1-binding protein 1 (TAX1BP1)	Q86VP1	W-type non-canonical	49-WVGI-52 141-LVV-143	23209807
TBC1 domain family member 25 (TBC1D25)	Q3MII6	W-type	136-WDII-139	21383079
TBC1 domain family member 5 (TBC1D5)	Q92609	W-type F-type	59-WEEL-63 787-FTIV-790	22354992
Tectonin beta-propeller repeat-containing protein 2 (TECPR2)	O15040	W-type	1408-WEVI-1411	26431026
Testis-expressed protein 264 (TEX264)	Q9Y6I9	F-type	273-FEEL-276	31006538
TNF receptor-associated factor 6 (TRAF6)	Q9Y4K3	Y-type	406-YISL-409	30806153
Transcription factor E2F3 isoform d (E2F3d)	O00716	Y-type	160-YSRL-163	30740539
Transitional endoplasmic reticulum ATPase (TER ATPase)	P55072	Y-type F-type	143-YRPI-146 163-FKVV-166	27561680
Translocation protein SEC62 (SEC62)	Q99442	F-type	363-FEMI-366	27749824
Tripartite motif-containing protein 5 (Trim5α)	Q0PF16	W-type (C- to N-terminus orientation)	193-ILDW-196	30282803
Tumor protein p53-inducible nuclear protein 1 (TP53INP1)	Q96A56	W-type	31-WILV-34	22470510
Tumor protein p53-inducible nuclear protein 2 (TP53INP2)	Q8IXH6	W-type	35-WLII-38	22470510
Ubiquitin-like modifier-activating enzyme 5 (UBA5)	Q9GZZ9	non-canonical	341-WGIEL-345	26929408 30990354
von Hippel-Lindau disease tutor suppressor (VHL)	P40337	Y-type	98-YPTL-101	30902965
WD repeat and FYVE domain-containing protein 3 (Alfy)	Q8IZQ1	F-type	3346-FIFV-3349	24668264
WD repeat-containing protein 81 (WDR81)	Q562E7	W-type Y-type	544-WIDL-547 578-YGVV-581	28404643

*For each interactor, we provide the UniProt ID, the LIR type, the LIR core sequence and the PMID of the corresponding scientific publications.*

The several variations around the LIR-binding mode suggest a binding promiscuity and adaptability of the hydrophobic groove of ATG8 proteins. These observations also imply that we have only scratched the surface of the known interactors for this class of proteins.

### Non-canonical LIR Motifs (CLIRs)

Non-canonical LIR motifs (CLIRs) do not fit the sequence requirements described so far. The binding modes of non-canonical LIRs are case-specific, and the atypical binding mode can depend on different structural determinants. The first CLIR was originally described as a LIR lacking the first aromatic Φ_1_ and consisting of aliphatic amino acids in the positions 2–4 of the motif, such as leucine or valine. The first CLIR refers to the XLVV core LIR motif of CALCOCO2 (also named NDP52) (von Muhlinen et al., 2012). CLIRs lacking the residue for interaction with HP1 are likely to depend on the adjacent amino acids for their binding affinity to the ATG8 proteins (von Muhlinen et al., 2012). In the CALCOCO2 CLIR, I133 is occupying the Φ_1_ position, but it is not able to fully occupy HP1 due to its small size. As a result, the side chain of I133 is partially solvent-exposed, and its mutation does not affect the binding. The LVV motif, on the contrary, forms an extended hydrophobic surface, which is matched on the surface of LC3C by part of the rim of HP2 and other surrounding residues (von Muhlinen et al., 2012).

UBA5, an E1-like ligase for the ubiquitin-like protein UFM1 harbors a promiscuous LIR motif (WGIELV) able to bind both UFM1 and ATG8 proteins (Habisov et al., 2016; Huber et al., 2019). In the complexes of UBA5 with GABARAP and GABARAPL2, the binding of V346 (X_6_) to the HP2 pocket is canonical-like, whereas I343 (X_3_), and L345 (X_5_) occupy simultaneously the HP1 pocket (Huber et al., 2019). Moreover, a partial structural rearrangement upon binding of UBA5 creates a new hydrophobic pocket called HP0, formed by the hydrophobic moieties of residues K46, K47, K48, V4, Y5, I32, and V33 of GABARAP, which accommodate the conserved W341 (Φ_1_). Mutations of the UBA5 LIR residues occupying HP1 and HP2 cause a moderate decrease in binding affinity, whereas the mutation of W341 to a non-aromatic amino acid dramatically reduces the binding, indicating that it is the most important residue for the interaction (Huber et al., 2019).

### Residues Flanking the Core LIR Sequence

The residues at the N- or C-terminal regions of the LIR core motif can interact with ATG8 proteins in different ways, contributing to stabilize the binding or providing specificity toward a certain class of ATG8 proteins. This highlights the importance of using long LIR constructs to study ATG8s-LIR interactions. Most of the available X-ray structures of ATG8s-LIR complexes are limited by the fact that the atomic coordinates of the residues flanking the LIR core motif are missing, making them a suitable case of study for molecular modeling. While the peptide flanking the LIR sequence is thought to be disordered in most cases, the presence of a C-terminal α-helix can allow a wider interaction surface and contribute to the higher binding affinity for some LIRs. More in general, recent studies suggest that ATG8s-LIR interactions extend up to 4–6 residues upstream and 8–10 residues downstream of the core motif. Salt bridges have been found to be a common mode of interaction between extended LIRs and human ATG8 proteins. However, hydrophobic interactions and hydrogen bonds in the LIR flanking regions can also contribute.

For instance, residues of FYCO1 at positions up to X_9_ are involved in substantial electrostatic and hydrophobic interactions with LC3A and B (Olsvik et al., 2015; Cheng X. et al., 2016). On the other hand, residues of the LIRs of ULK1, ATG13, or PCM1 located upstream (in positions X_–1_ and X_–2_) and downstream (X_5_-X_11_) of the core sequence contribute to important interactions with GABARAP (Wirth et al., 2019). Our group also demonstrated, using MD simulations validated by experimental mutagenesis, the importance of the acidic stretch N-terminal to the core motif of PCM1 (DEED) simultaneously promoting the interaction with a lysine cluster and disfavoring the interaction with the negatively charged residue E8 of GABARAP (Holdgaard et al., 2019).

As previously mentioned, the presence of acidic amino acids (i.e., aspartate or glutamate) at the N- or C-terminal regions of the core LIR motif has been reported for several complexes. This is due to the possibility of interaction with the positively charged residues of ATG8 proteins in the surroundings of the LIR binding site. Supporting this notion, alanine substitutions of the SQSTM1 LIR residues D337 and D338 (X_–__3_ and X_–2_, respectively), which interact with R10 and R11 of LC3B, almost abolish the binding (Pankiv et al., 2007; Ichimura et al., 2008). Similarly, negatively charged residues in positions X_–1_ and X_–2_ are important for the binding of PCM1 and ULK1 to LC3s, as their removal significantly reduces the binding (Holdgaard et al., 2019; Wirth et al., 2019). Other complexes, featuring similar electrostatic interactions due to charged residues flanking the LIR motif, include: (i) ATG14, for which the mutation of aspartate and glutamate residues immediately preceding the motif drastically reduces the binding to ATG8 proteins (Birgisdottir et al., 2019); (ii) Alfy, in which D3344 (X_–2_) interacts with K24 of GABARAP (Lystad et al., 2014); (iii) ATG4B, in which four N-terminal acidic residues (X_–4_-X_–1_) interact with H9, K20, K46, R47, and K48 of GABARAPL1 (Skytte Rasmussen et al., 2017) and, (iv) FYCO1, where two negatively charged residues in X_–3_ and X_–4_ position interact with R10 of LC3B in a tripartite salt-bridge network (Olsvik et al., 2015). Similar electrostatic interactions have also been identified in the X-ray structures of the following complexes: LC3A-RETREG1 (Khaminets et al., 2015), LC3B-FUNDC1 (Kuang et al., 2016), GABARAPL1-NBR1 (Rozenknop et al., 2011), LC3C-CALCOCO2 (von Muhlinen et al., 2012), making it a common mechanism by which LIR-containing proteins attain a stronger binding.

## Specificity of Different ATG8 Family Members in LIR Recognition

The presence of different ATG8 orthologs, which interact with different LIR-containing proteins, raises the question about the specificity of these interactors toward each member of the ATG8 family or a subset of them, which may be intertwined with the role of these proteins in autophagy (Kriegenburg et al., 2018).

The structural mechanisms of the interaction between ATG8 proteins and several LIR motifs have gained attention over the last decade, but we are still missing a comprehensive view of the determinants of their binding specificity. They might be case-specific and acquired by a multitude of diverse structural mechanisms.

In the last few years, at least three different studies addressed this open question (Rogov et al., 2017a; Atkinson et al., 2019; Wirth et al., 2019), so far marginally treated in studies dedicated to individual interactors. These more comprehensive works either focused on the identification of the structural determinants of the specificity for one subfamily (Rogov et al., 2017a), on a subset of functionally related LIR-containing interactors (Wirth et al., 2019) or more broadly engaged in the investigation of the specificity of representative LIRs (Atkinson et al., 2019).

Several techniques have been employed to investigate the specificity of ATG8s binding partners (Johansen et al., 2017; Weiergräber et al., 2017; Atkinson et al., 2019; Rasmussen et al., 2019), including both biochemical and biophysical assays. Some examples include pull down, co-immunoprecipitation, peptide arrays, isothermal titration calorimetry, surface plasmon resonance or, FRET. Moreover, structural biology approaches, like X-ray crystallography and NMR, often accompanied by MD simulations, have been unveiling the underlying molecular mechanisms. The results collected so far indicate that the specificity of LIR-containing ATG8 interactors can be summarized in five categories: those with a preference for (i) LC3A/B; (ii) LC3C only; (iii) LC3C and the GABARAP subfamilis, (iv) only GABARAP proteins and, (v) nonspecific binders (i.e., promiscuous interactors). A sixth category includes those interactors for which there are not enough data available to determine a preferential binding.

In general, LIR motifs displaying non-canonical features seem to bind preferentially to either LC3C or GABARAPs, as seen for CALCOCO2 (von Muhlinen et al., 2012) and UBA5 (Habisov et al., 2016; Huber et al., 2019). In this group, the specificity of canonical motifs highly depends on the residues flanking the core LIR (Atkinson et al., 2019) or the residues between the Φ_1_ and Ψ_4_ positions (Rogov et al., 2017a). In particular, acidic residues at both C- and N-terminal regions with respect to the core motif seem important for binding LC3A and LC3B. This was suggested to be related to interactions with H57, which is only conserved in LC3A/B and interacting with the C-terminal acidic residues of the LIRs (Olsvik et al., 2015; Cheng X. et al., 2016; Atkinson et al., 2019). However, the scenario is not straightforward, as negatively charged residues upstream of the core motif can also be important for binding to the GABARAP subfamily. Moreover, different charge distributions in the surroundings of the LIR binding region contribute to the complexity of this scenario. All these factors may promote a different conformation of the LIR in the ATG8 pocket (Holdgaard et al., 2019). Moreover, some LIR C-terminal extensions can impair the binding to LC3 proteins, as happens for the LIR motif of ULK1, where the concerted effect of a methionine within the core LIR and a proline C-terminal to the motif is key in shifting the specificity toward the GABARAP subfamily (Wirth et al., 2019). The GABARAP subfamily might also tolerate the absence of a C-terminal extension of the LIR, as seen in TP53INP1 (Atkinson et al., 2019) and ATG4C (Skytte Rasmussen et al., 2017; Atkinson et al., 2019).

**FIGURE 5:**
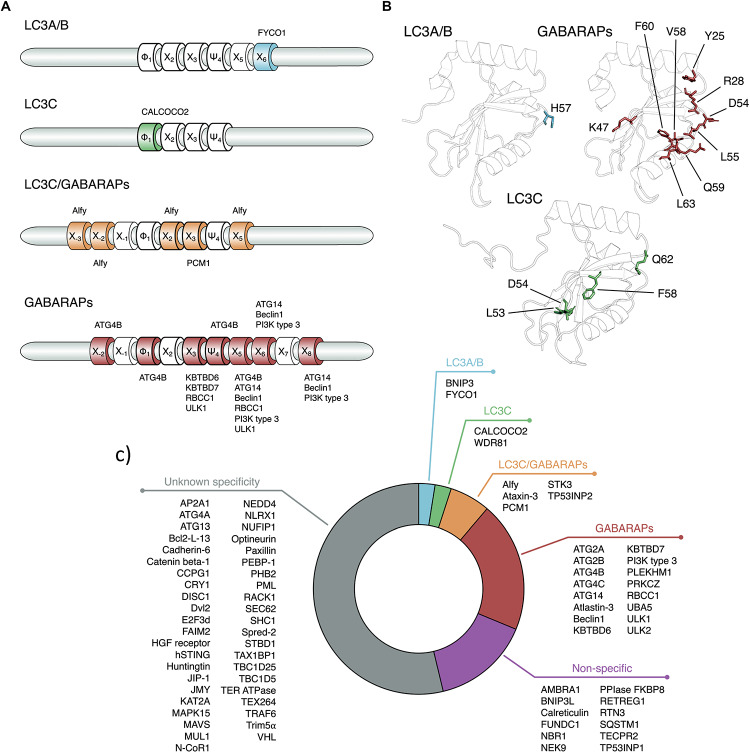
Specificity of the LIR-containing proteins for different human ATG8 subfamilies. **(A)** We illustrate the LIR sequences, highlighting the positions important in determining the LIR specificity. The core LIR positions are drawn with a thicker border. The positions determining the specificity of certain LIR-containing proteins are annotated with the corresponding protein names. **(B)** The residues important in determining the specificity for a protein/subfamily are displayed as sticks on the 3D structure of each ATG8 protein and labeled. We used the following PDB entries: 3VTU for LC3A/B (LC3B), 1KJT for GABARAPs (GABARAP), and 2NCN for LC3C (Bavro et al., 2002; Rogov et al., 2013; Krichel et al., 2019). **(C)** Specificity of known LIR-containing proteins. The color-coding of **(A,B)** is consistent with the one defined in **(C)**.

For the GABARAP subfamily, residues important for subfamily-specific interactions include E8, H9, (Holdgaard et al., 2019), K24 (Lystad et al., 2014; Wirth et al., 2019), Y25 (Genau et al., 2015; Holdgaard et al., 2019; Wirth et al., 2019), R28 (Skytte Rasmussen et al., 2017; Wirth et al., 2019), K/R47 (Skytte Rasmussen et al., 2017; Huber et al., 2019), D54 (Lystad et al., 2014; Birgisdottir et al., 2019), L55 (Birgisdottir et al., 2019), Q59 (Wirth et al., 2019), and F60 (Birgisdottir et al., 2019). The different specificity groups are described below and summarized in Figure 5.

### LC3A/B

FYCO1 binds to all the ATG8 human orthologs (Pankiv et al., 2010; Olsvik et al., 2015), but shows a preference for LC3A and LC3B (Olsvik et al., 2015; Cheng X. et al., 2016; Atkinson et al., 2019), partially due to a specific interaction formed between D1285 at position X_6_ of the LIR motif and H57 in the ATG8 protein (Olsvik et al., 2015). BNIP3 interacts with higher affinity with LC3B (Hanna et al., 2012; Zhu et al., 2013), weakly with GABARAPL2 (Zhu et al., 2013) and not with GABARAP (Hanna et al., 2012) or GABARAPL1 (Zhu et al., 2013).

### LC3C

CALCOCO2 selectively binds to LC3C (von Muhlinen et al., 2012) via its non-canonical LIR motif, as previously described (see section “Non-canonical LIR Motifs (CLIRs).” Interestingly, mutating the isoleucine immediately N-terminal to the LVV motif (I133) to a tryptophan, thus restoring a canonical motif, increases the binding of CALCOCO2 toward the other ATG8 human orthologs (von Muhlinen et al., 2012). WDR81 also selectively interacts with LC3C, and this interaction is abrogated when either one of the two identified LIRs is mutated (Liu et al., 2017).

### LC3C and GABARAPs

The LIR motif of Alfy interacts selectively with the GABARAP subfamily, and only weakly with LC3C (Atkinson et al., 2019). An alanine substitution of I3347 (position X_2_ of the LIR) weakens the binding to LC3C, indicating the possibility for a hybrid canonical/non-canonical LIR (Lystad et al., 2014). At the structural level, the specificity seems to be due to the formation of interactions between K3343, D3344 of Alfy (positions X_–3_ and X_–2_ of the LIR), and K24, Y25 of GABARAP. An interaction between Y3351 (Alfy, position X_5_) and D54 (GABARAP) can also contribute. Indeed, if K24 and Y25 in GABARAP (K32 and F33 in LC3C) are replaced by the glutamine and histidine typical of LC3B, interactions with K3343 and D3344 are lost. In contrast, the substitution of D54 with histidine (H57 in LC3B) may cause severe steric hindrance.

PCM1 also binds to the GABARAP subfamily and LC3C and weakly to LC3A/B (Holdgaard et al., 2019; Wirth et al., 2019). However, the substitution of the lysine in position X_3_ (K1965) with isoleucine, leucine, valine or phenylalanine dramatically increases the binding affinity toward LC3B. Conversely, the mutation to lysine of the residues at position X_3_ in the LIRs of ATG4B, FUNDC1 or PPIase FKBP8 decreases the binding to LC3A/B/C (Wirth et al., 2019). Swapping mutations on the ATG8 interactor also confirmed the specificity of PCM1 (Holdgaard et al., 2019). In this context, residues of GABARAP proteins suggested important for the specificity of PCM1 are: (i) Q59 (conserved in GABARAPs and LC3C) that decreases the binding affinity of PCM1 toward GABARAP when mutated to the glutamate of LC3A/B (Wirth et al., 2019) and vice versa; (ii) Y25, which abolishes the binding of PCM1 to GABARAP when mutated to the corresponding histidine of LC3B (i.e., H27) and vice versa (Holdgaard et al., 2019); and (iii) E8 and H9 that weaken the binding of PCM1 to GABARAP if mutated to the corresponding arginines of LC3B (Holdgaard et al., 2019). Surprisingly, mutating L55 in GABARAP to the corresponding valine in LC3s does not have a marked effect on the binding of both PCM1 and ULK1 (Wirth et al., 2019), differently from what observed for Beclin1, ATG14, and PI3K type 3 (Birgisdottir et al., 2019). This suggests a different binding mode for the residues located C-terminally to the core LIR in different proteins.

Ataxin-3 preferentially binds GABARAP and LC3C with either of its two recently identified LIR motifs. A weak binding of Ataxin-3 has also been detected with LC3A but its dependence on the LIR motif remains to be confirmed. Not only LC3B but also GABARAPL1/L2 seem not to bind Ataxin-3 (Herzog et al., 2019). TP53INP2 also shows a higher binding affinity for LC3C and GABARAPs compared to LC3A/B, but the molecular details underlying this preferential binding are still unclear (Atkinson et al., 2019).

The serine/threonine kinase STK3 preferentially binds LC3C and GABARAP, even if it was shown to bind all ATG8 proteins (Shrestha et al., 2020). The interaction has been confirmed to be mediated by a noncanonical LIR motif, resembling that of CALCOCO2, for LC3C and GABARAP (Shrestha et al., 2020).

### GABARAPs

Cellular experiments with *Atg14* knock-out cells reconstructed with ATG14 wild-type or binding-deficient mutants confirmed the preferential binding of both ATG14 and Beclin1 for the GABARAP subfamily (Birgisdottir et al., 2019). The authors also showed that an intact ATG14 LIR is required for the phosphorylation of S29 on ATG14 itself and for an effective binding of Beclin1 to the GABARAP subfamily. However, the structural details of both these mechanisms remain to be elucidated.

ATG4B interacts with LC3A/B/C and GABARAP/L1/L2, and the simultaneous alanine mutation of the two core residues of the LIR (F388 and L391) seems to have a greater impact on the binding to the LC3 subfamily members than to the GABARAP proteins (Skytte Rasmussen et al., 2017). The GABARAP residues R28 and R47 are replaced by lysine in the LC3 subfamily, and this may contribute to the lower binding affinity of the ATG4B LIR toward LC3 proteins. Indeed, the smaller side chain of lysine may not engage in interactions with L391, S392, and E386 as efficiently as an arginine (Skytte Rasmussen et al., 2017). The ATG4B LIR motif is identical to the LIR of ATG4A, which has been shown to interact with both LC3B and GABARAP. On the other hand, another member of the ATG4 family, ATG4C, which has a different core LIR sequence (FVLL) and no C-terminal extension, displays a preferential binding for GABARAP over LC3B (Skytte Rasmussen et al., 2017).

Beclin1 and PI3K type 3, members of the PtdIns3k complex I, also display a clear preference for binding the GABARAP subfamily (Birgisdottir et al., 2019). However, their LIR core sequences deviate from the recently proposed GABARAP-interaction motif (GIM) Φ_1–_[V/I]_2_-X_3–_V_4_ (Rogov et al., 2017a). The reason for this preference might reside in differences in the HP2 pockets between the GABARAP and the LC3 subfamily. In fact, residues L55 and F60 of GABARAP proteins are replaced by valine and leucine in LC3A/B, whose shorter side chains may not be able to properly engage the side chain of residues in positions X_5_, X_6_, and X_8_ of the LIR motif. The fact that the LIRs of PI3K type 3 and ATG14 are also able to bind LC3C, where leucine and phenylalanine are conserved (i.e., L53 and F58), support the structural hypothesis mentioned above. Furthermore, D54 in the GABARAP subfamily is involved in interactions with residues in positions X_6_ and X_8_ and replaced by the bulkier H57 of LC3B, which may cause severe steric clashes.

The importance of the C-terminal residues for binding specificity is further underlined in the LIR of ULK1, where the removal of the seven residues C-terminal of the core LIR broadens the LIR specificity to LC3 proteins (Wirth et al., 2019). M359 (X_3_) and P361 (X_5_) are likely to prevent the wild-type ULK1 LIR motif from binding LC3 proteins, as supported by the fact that M359I and P361D mutations of ULK1 increase the binding toward LC3A/B more than other mutations tested in the same work. This suggests that a longer side chain (for M359I) or a charged residue (for P361D) could provide more favorable interactions of the LIR with LC3 proteins. More studies are needed to elucidate the underlying structural mechanisms. Moreover, the proline at X_5_ in ULK1 and RBCC1 seems to be tolerated only by GABARAP subfamily members, while its mutation to an aspartate slightly increases the binding to LC3s. Indeed, the cyclic nature of the proline side chain might pose geometric constraints unfavorable for binding LC3s over GABARAP proteins (Wirth et al., 2019). The binding of ULK1 and RBCC1 (Alemu et al., 2012) to LC3B also considerably increases when the residue at position X_3_ of the core motif is mutated to a hydrophobic one (i.e., I, L,V, or F). On the other hand, introducing a lysine at this position in FUNDC1 and PPIase FKBP8 impair their binding to LC3 proteins, and shifts the binding specificity of ATG4B toward GABARAP proteins (Wirth et al., 2019). ULK2 also preferentially interacts with GABARAPs (Alemu et al., 2012), even though the determinants of such specificity have not been investigated in detail yet.

The non-canonical LIR of UBA5 also shows a clear preference for the GABARAP subfamily, displaying no binding to LC3B and LC3C and weak binding to LC3A (Habisov et al., 2016). Notably, the β2–β3 loop and the end of the β2 strand, where the GABARAP-specific K/R47 flanks two conserved lysines, have been proposed as determinants for GABARAP specificity of UBA5 (Huber et al., 2019).

KBTBD6 and KBTBD7 display preferential binding to GABARAPs, although they are also able to bind LC3 proteins (Genau et al., 2015). This preference may be explained by additional interactions made by R670/Q670 (X_3_) and V671 (X_4_) with Y25, V58, F60, and L63 of GABARAP. In particular, the hydrogen bond formed by the guanidinium group of R670 in KBTBD6 (Q670 in KBTBD7) and the hydroxyl group of GABARAP Y25 could stabilize the conformation of V671 and regulate the orientation of the entire LIR motif in the binding pocket. Indeed, this hydrogen bond cannot be formed by the corresponding residue H27 in LC3A/B.

The LIR of PLEKHM1 has been initially shown to interact with all ATG8 human orthologs (McEwan et al., 2015). Nevertheless, more detailed studies with both biochemical and biophysical assays (Rogov et al., 2017a) pointed toward a tighter binding of PLEKHM1 to the GABARAP subfamily.

The LIR of the kinase PRKCZ preferentially binds to the GABARAP subfamily as well. It also binds LC3B very weakly, while no interaction has been found with LC3C (Shrestha et al., 2020). Even if the interaction with LC3B was reported as very weak both *in vitro* and *in vivo*, LC3B can efficiently co-immunoprecipitate PRKCZ from cell extracts, suggesting that either post-translational modifications are necessary for the binding or the association is indirect (Shrestha et al., 2020).

The LIR motifs of the autophagy-related proteins ATG2A and ATG2B have also been shown to interact preferentially with GABARAP and GABARAPL1 (Bozic et al., 2020). ATG2A and ATG2B did not interact with LC3B/C or GABARAPL2, and only weakly with LC3A in co-immunoprecipitation assays (Bozic et al., 2020).

### Non-specific LIRs

TECPR2 binds to LC3s and GABARAPs with similar affinity, the strongest binders being LC3B/C and GABARAP, and the weakest one being GABARAPL2 (Stadel et al., 2015). AMBRA1 also binds to all the ATG8 proteins, even though a slight preference toward GABARAP has been observed (Di Rita et al., 2018).

PPIase FKBP8 also binds to both LC3 and GABARAP proteins (Wirth et al., 2019). Two distinct LIR motifs are able to mediate this interaction, as proved for the binding to LC3B (Yamashita et al., 2019). The LIR located more C-terminally seems to mediate also the interaction of PPIase FKBP8 with the mitochondrial transmembrane protein OPA1 (Yamashita et al., 2019). The protein kinase NEK9 is also able to bind all ATG8 proteins with similar affinities (Shrestha et al., 2020).

The LIR motif of BNIP3L (also called NIX) also displays a non-specific binding pattern. It interacts with both LC3 and GABARAP proteins, whereas LC3B is a weaker binder for BNIP3L. However, its interaction with both LC3A and LC3B is drastically enhanced when two serine residues located N-terminal to the core LIR (S34 and S35) are phosphorylated or substituted by phosphomimetic residues (Rogov et al., 2017a). For LC3B, this is probably due to additional interactions between the side chains of the phosphorylated serines and those of R11 and K51, as also demonstrated for the LIR motif of optineurin (Rogov et al., 2013).

RETREG1 has been shown to interact with LC3A, LC3B, and GABARAPL2 (Khaminets et al., 2015), indicating no binding specificity toward either the LC3 or the GABARAP subfamily. NBR1 and SQSTM1 also seem to be non-specific, as they interact with all the LC3 and GABARAP proteins (Pankiv et al., 2007; Ichimura et al., 2008; Shvets et al., 2008; Kirkin et al., 2009; Rozenknop et al., 2011; Goode et al., 2016; Atkinson et al., 2019). Nevertheless, in SQSTM1, R10 and R11 of LC3A/B may be the structural determinants for a more specific binding toward LC3s, since mutating these residues to the corresponding ones in GABARAP (E8 and H9) significantly reduces the binding of SQSTM1 to LC3B, while mutating E8 and H9 to arginines in GABARAP enhances its interaction with SQSTM1 (Wirth et al., 2019). The LIR of calreticulin also binds GABARAP, LC3A and LC3B (Mohrlüder et al., 2007; Thielmann et al., 2009; Yang et al., 2019), suggesting binding promiscuity. FUNDC1 interacts with LC3A/B and GABARAP/L2 (Liu et al., 2012; Chen et al., 2014; Wu et al., 2014; Kuang et al., 2016; Lv et al., 2016), but the binding to GABARAPL1 and LC3C has not been investigated so far.

RTN3 (Grumati et al., 2017) and TP53INP1 (Atkinson et al., 2019) interact with all the human ATG8 variants. In RTN3, only a deletion of all its six LIR motifs was able to completely disrupt the binding with both the ATG8 subfamilies (Grumati et al., 2017), indicating that all the motifs are functional, they can mediate the binding and are likely to compensate for each other’s loss. In TP53INP1, the deletion of the three negatively charged residues N-terminal to the core LIR reduces the affinity of the LIR for LC3A/B. In contrast, the same deletion has only modest effects on the binding with other ATG8 proteins. Similarly, the deletion of the region upstream of the core motif has a limited effect on the binding with LC3C and GABARAP proteins, indicating a stronger dependence on the N-terminal residues for the binding with LC3A/B. Removing the aspartate in position X_8_ and the cysteine at position X_10_ of the LIR reduces the interaction with both LC3 and GABARAP subfamilies, but with a more pronounced effect for LC3A/B in the case of the cysteine deletion (Atkinson et al., 2019). This may be due to the loss of interactions between the cysteine and I66 and R70 in LC3B. Specific binding to GABARAP proteins can also be achieved when no residues C-terminal to the core LIR are present and only the three N-terminal acidic residues and the core motif are retained. This result confirms that the interaction with GABARAP subfamily members can tolerate amino acid truncation at a much higher degree than the recognition by the LC3 subfamily (Atkinson et al., 2019).

### Unknown Specificity

The majority of the ATG8 interactors identified so far have been tested for their LIR-mediated interaction with LC3B, not allowing to draw any conclusions about their preferential binding to other ATG8 proteins. Moreover, an interaction between a LIR-containing protein and an ATG8 protein is often reported without confirming that the binding is mediated by the LIR region and affect the binding specificity using site-directed mutagenesis. For instance, NEDD4 has been identified in a proteomic study as an interactor of both LC3 and GABARAP subfamilies (Behrends et al., 2010), but only its interaction with LC3B has been validated (Qiu et al., 2017; Sun et al., 2017). The same holds for E2F3d, which is able to interact with both LC3A/B and GABARAP/L2, but for which only the binding to LC3B has been confirmed as LIR-mediated (Araki et al., 2019). Dvl2 binds LC3B and GABARAP, but not GABARAPL2 and only the LC3B interaction has been confirmed as LIR-dependent by mutagenesis (Gao et al., 2010). WDR81 interacts with LC3C and weakly with GABARAP/L1, whereas it does not bind to LC3A and GABARAPL2 but only the LIR-dependent interaction with LC3C has been confirmed (Liu et al., 2017).

AP2A1 (Tian et al., 2013), Bcl2-L-13 (Murakawa et al., 2015), JIP-1 (Sandilands et al., 2011), SEC62 (Fumagalli et al., 2016), paxillin (Sharifi et al., 2016), SHC1 (Onnis et al., 2018), NUFIP1 (Wyant et al., 2018), NLRX1 (Zhang et al., 2019), JMY (Coutts and La Thangue, 2015), VHL (Kang et al., 2019), TRAF6 (Wu et al., 2019), PHB2 (Wei et al., 2017), PEBP-1 (Noh et al., 2016), optineurin (Rogov et al., 2013), TEX264 (Chino et al., 2019), Trim5α (also known as MURF2B) (Pizon et al., 2013), HGF receptor (also called MET) (Huang et al., 2019), KAT2A (Ouyang et al., 2019), and Fas-apoptotic inhibitory molecule 2 (FAIM2) interact with LC3B via a LIR motif (Jeeyeon et al., 2020). These LIRs were not tested for binding with other ATG8 proteins. For SHC1 the interaction has been confirmed to be LIR-mediated with LC3B-II, but not for the unprocessed form of LC3B (Sun et al., 2016; Onnis et al., 2018).

CSRP3 (Rashid et al., 2015) and FHL1 (Han et al., 2020) have been recently found to interact with LC3B as well, but the interaction remains yet to be confirmed to be LIR-dependent.

ATG13 has been found interacting with all the LC3 proteins (with an appreciable preference for LC3A/C over LC3B, whose structural details remain yet to be determined) but has not been tested with the GABARAP subfamily (Suzuki et al., 2014).

In other cases, the interaction with both LC3B and at least GABARAP has been tested to recapitulate the specificity toward one of the two subfamilies. In these cases, it will be beneficial to investigate the binding to LC3C and GABARAPL2, which often deviates from the preferences of their respective subfamily. CCPG1 (Smith et al., 2018), catenin beta-1 (Petherick et al., 2013; Rogov et al., 2017a), ATG4A (Skytte Rasmussen et al., 2017), and MAPK15 (Colecchia et al., 2012) belong to this group. Moreover, FLCN has also been shown to interact with both LC3B and GABARAP with a preference for the latter. Both these interactions remain to be confirmed as LIR-dependent (Dunlop et al., 2014). TBC1D25 (also called OATL1) interacts with LC3B, GABARAP and GABARAPL2, but, in this case, only the interaction with GABARAP has been confirmed as LIR-mediated (Itoh et al., 2011).

STDB1 interacts with GABARAPL1 via its LIR motif (Jiang et al., 2011), but the other ATG8 proteins remain to be tested. On the contrary, the non-canonical LIR motif of TAXBP1 has only been validated for the interaction with LC3B and LC3C (Tumbarello et al., 2015). A strong binding between TAXBP1 and GABARAPL1/L2 has also been detected, along with a weaker one with GABARAP and LC3A, but their dependence on a LIR motif remains to be verified (Tumbarello et al., 2015). A MUL1 (Mulan)-Ube2E3 heterodimer also interacts with GABARAP but not with LC3B, even if the interaction with other LC3 and GABARAP proteins remains to be probed (Ambivero et al., 2014). Instead, N-CoR1 seems to preferentially bind GABARAP subfamily members rather than LC3B, but the relevance of the LIR motif in such interaction has only been proved for GABARAP (Saito et al., 2019). DISC1 (Wang et al., 2019), MAVS (Sun et al., 2016; Cheng et al., 2017), and hSTING (Liu et al., 2019) interact with a member of the LC3 family, but no information about the specific binding partner was provided.

### Proteins With Multiple LIRs

For a group of LIR-containing proteins, two or more LIR motifs have been found to interact with LC3B and validated by experimental mutagenesis, i.e., TER ATPase (also named VCP), PML, RACK1, Spred-2 and CRY1 (He et al., 2014; Cheng M. et al., 2016; Guo et al., 2016; Jiang et al., 2016; Toledo et al., 2018). In the cases of PML and RACK1, the C-terminal motifs seem to play the most important role in the interaction (He et al., 2014; Cheng M. et al., 2016). A secondary LIR has also been reported in PCM1 for the interaction with GABARAP, which is likely to account for the remaining binding activity when the most important LIR is mutated (Joachim et al., 2017). The KXD1 subunit of BORC has four putative LIRs. The deletion of all of them impairs its interaction with LC3B, but single contributions to the binding have not been yet investigated (Jia et al., 2017). Similarly, the deletion of the two LIR motifs found in Syntaxin-17 impairs its binding to both LC3B and GABARAP. However, the contribution of the single LIRs to the interaction is yet to be elucidated (Kumar et al., 2018).

Huntingtin interacts with both LC3B and GABARAPL1, but the specific LIR(s) responsible for the interaction has (have) not been identified yet (Ochaba et al., 2014). A mutation in W3037 of huntingtin, predicted by iLIR as occupying the Φ_1_ position (Jacomin et al., 2016), does not impact the binding of full-length huntingtin with LC3B and GABARAPL1, but impairs the binding of a shorter construct of huntingtin (aa 2416-3144) to GABARAPL1. This result suggests both that this LIR is functional and that other LIR motifs in the longer construct may compensate for the mutation (Ochaba et al., 2014).

The binding of TBC1D5 to human ATG8s is also dependent on multiple LIRs. TBC1D5 interacts with LC3A, LC3B, and GABARAPL1 through its two LIRs (with the major contribution coming from the most C-terminal LIR), displaying no preference for one of the two subfamilies (Popovic et al., 2012). Cadherin-6 was predicted to contain two LIRs, one of which was found to mediate its interaction with GABARAP. In contrast, no interaction was detected with GABARAPL2 or LC3 proteins in two-hybrid yeast assays (Gugnoni et al., 2017), suggesting another candidate for more detailed studies. Multiple LIR sequences in multi-domain proteins are a class deserving further investigation, which well fits within the definition of SLiMs, where the repetition of a motif in the same protein sequence increases the binding affinity and avidity of the interaction (Davey et al., 2012). Multiple motifs could assist the formation of larger complexes in the proximity of autophagy membranes, where the same protein is engaging multiple ATG8 proteins simultaneously through different LIRs along its sequence.

More efforts and data on site-directed mutagenesis of the residues in the core LIR and in the flanking regions, along with information on the effect of swapping mutations, will be needed to unveil the preferences of the known LIRs. A convenient experimental approach could be based on peptide arrays for a first high-throughput screening, followed by isothermal titration calorimetry on selected candidates (Klionsky et al., 2016; Johansen et al., 2017; Rasmussen et al., 2019).

Modeling and simulations can also help in the quest for determinants of the specificity of LIR-containing proteins. These computational techniques have the advantage to provide insights into the related structural mechanisms. We have already cited some of these studies above (Di Rita et al., 2018; Aykac Fas et al., 2019; Holdgaard et al., 2019; Jatana et al., 2019) in section “Structural Dynamics of ATG8 Family Members.”

## Post-Translational Modulation of the LIR-ATG8s Interaction

Post-translational modifications, such as phosphorylation, may also play a role in tuning the specificity or binding affinity of LIR-mediated interactions. This has been shown, for example, for AMBRA1 (Di Rita et al., 2018), PI3K type 3 (Birgisdottir et al., 2019), BNIP3L (Rogov et al., 2017b), HGF receptor (Huang et al., 2019), and optineurin (Rogov et al., 2013).

The modulation of the ATG8s-LIR peptide binding through phosphorylation has been investigated in several cases, some of them including structural studies. As mentioned in the previous sections, many complexes feature acidic or phosphorylatable residues in the vicinity of the core LIR motif that contribute to the binding to the ATG8 proteins through electrostatic interactions. These residues are usually upstream with respect to the core LIR motif, and they consist of one or more phosphorylatable residues. In the studies carried out so far, the molecular mechanism and the determinants of binding for phosphorylated LIR motifs have been investigated using phosphorylated peptides or phosphomimetic mutations, namely mutations to acidic residues to mimic the effect of phosphorylation, such as the substitution of the phosphorylatable residue with aspartate or glutamate.

For the members of the autophagy class III phosphatidylinositol 3-kinase complex (PtdIns3k complex I), namely PI3K type 3, ATG14, and Beclin1 (Birgisdottir et al., 2019), phosphomimetic mutations increase the binding affinity for ATG8 proteins. S93E and S96E substitutions in Beclin1 enhance the binding threefold for GABARAP and GABARAPL1, fivefold for LC3A and eightfold for LC3C. For PI3K type 3, mutations of S244 and S249 were found to increase binding 17-fold for GABARAP, 19-fold for GABARAPL1 and 15-fold for LC3C. The S249E variant of the LIR of PI3K type 3 variant displayed broadened specificity and bound also LC3B. The structural determinants of this enhanced binding were studied using X-ray crystallography and solving the 3D structures of the complexes GABARAP-PI3K type 3 S249E (X_–1_) and GABARAP-Beclin1 S96E (X_–1_). While the binding mode was found to be similar between the wild-type and the mutant, E96 interacted favorably with K46, thus explaining the stronger interaction; similarly, E249 formed a salt bridge with K48 in the GABARAP-PI3K type 3 complex (Birgisdottir et al., 2019).

Another phospho-regulated LIR is that of the autophagy receptor BNIP3L (Rogov et al., 2017b). In this case, the canonical core LIR motif is preceded by two serine residues with two more downstream (LNSSWVELPMNSSN). S34 (X_–2_) and S35 (X_–1_) are conserved between BNIP3L and its homolog BNIP3. The mutations S34E and S35E increase the binding to LC3A/B in an additive manner, so that the mutation of both increases the affinity for LC3B by about 100-fold. Structural and biophysical characterization of the phosphomimetic double mutant S34E/S35E in complex with LC3B has shown that E34 interacts with the R10 and K51 residues of LC3B; furthermore, the side chain of R11 changes orientation and lies in the proximity of E34. In fact, mutants of LC3B that abolish the positive charge of residues involved in the interaction, such as R11A or K51A on LC3B, have a reduced affinity for the phosphomimetic mutant. The fact that the R11A substitution has been proven more effective than R10A in removing the binding is compatible with the dynamic nature of the region of the protein surrounding such residues.

Another example is the autophagy receptor optineurin, in which five serine residues precede the LIR motif (SSGSSEDSFVEI) (Rogov et al., 2013), with one of them (S177) positioned directly before the Φ_1_ residue. Phosphorylation of S177 causes a marked enhancement in binding affinity between optineurin and LC3B with small additive effects from the other phosphorylations. NMR and ITC studies of the complexes, featuring either a phosphomimetic mutant harboring five serine-to-glutamate mutations or a fully phosphorylated variant, shed light on the structural determinants of the interaction. R11 in LC3B was found to be the residue most important for the interaction with E177, which, in turn, caused other long-range effects in the hydrogen-bond network of the ATG8 protein. The presence of a phenylalanine instead of the tryptophan in position Φ_1_ of this LIR may be necessary for a system switchable through phosphorylation (Rogov et al., 2013).

FUNDC1 represents an atypical example of modulation through phosphorylation. FUNDC1 LIR contains a canonical Y-type LIR motif (YEVL) and the phosphorylation occurs on the Φ_1_ residue (Y18). Phosphorylations on Y18 or S13 (X_–5_) by the CSNK2/CK2 kinase and Src, respectively, can abolish the interaction of FUNDC1 with LC3B. FUNDC1 dephosphorylation during hypoxia allows the binding of ATG8 proteins and the induction of mitophagy. In fact, phosphorylation of Y18 (but not of S13 alone) significantly reduces the affinity of FUNDC1 for LC3B. This was corroborated by NMR studies and by the crystallographic structure of the unphosphorylated variant (Kuang et al., 2016). The phosphorylation of S17 (X_–1_) increases threefold the affinity between LC3B and FUNDC1, promoting interactions with K49 in LC3B. Indeed, K49A and especially K49E substitutions significantly reduce the binding affinity, thus underlining the importance of the electrostatic interaction. Interestingly, the interaction with the phospho-residue also changes the conformation of K49 (Lv et al., 2016).

The phosphorylation of a serine upstream of the core LIR sequence of the autophagy receptor AMBRA1 (S1043, position X_–6_) has been investigated integrating experimental and computational techniques (Di Rita et al., 2018). ITC and NMR experiments have demonstrated that S1043 phosphorylation increases the binding affinity of the AMBRA1 LIR for ATG8 proteins (Di Rita et al., 2018). Furthermore, chemical shift perturbation analyses by NMR showed that the AMBRA1 LIR binds to LC3B in a canonical manner and that the S1043 phosphorylation enhances this interaction. In the same study, our MD simulations highlighted that the structural flexibility of the AMBRA 1 LIR in the binding pocket of LC3B decreases upon S1043 phosphorylation, rescuing a flexibility profile similar to the one of the canonical SQSTM1 LIR in complex with LC3B. This suggests that phosphorylation of S1043 may compensate for the low binding affinity of the unphosphorylated LIR of AMBRA1. Moreover, the analysis of the MD conformational ensemble using the Protein Structure Network paradigm (Tiberti et al., 2014; Papaleo, 2015) suggests that the stabilization induced by the S1043 phosphorylation may be associated with an increased number of electrostatic interactions (both local and long-range) formed by the AMBRA1 LIR with LC3B. Such interactions involve positively charged residues in LC3B (such as R10, R11, K49, K51, R69, and R70) already shown to interact with the negatively charged (or phosphorylatable) residues found upstream of other LIR motifs.

Phosphomimetic mutations of two serines upstream of the core LIR sequence (S78 and S82, positions X_–5_ and X_–1_) also enhance the interaction of the mitophagy regulator BNIP3 with both LC3B and GABARAPL2. S78 phosphorylation displays the most substantial contribution to the binding affinity toward the two proteins. The results suggest a broadened specificity of BNIP3 induced by phosphorylation, and this modulation might be important for the coupling of BNIP3-targeted mitochondria to the autophagosomal membrane (Zhu et al., 2013).

The binding of paxillin (Sharifi et al., 2016) and HGF receptor (Huang et al., 2019) LIRs to LC3B may also be regulated by phosphorylation, since the Φ_1_ residues are tyrosines which are known phosphorylation sites. The kinase involved in the Φ_1_ phosphorylation of paxillin is Src and the expression of a constitutively active form of Src increases the interaction of the protein with LC3B (Sharifi et al., 2016), supporting a regulatory phosphorylation at Φ_1_. In the HGF receptor, dephosphorylation of the tyrosines at positions Φ_1_ and X_2_ of its LIR motif (Y1234/1235) has been associated with autophagy activation and survival in liver cancer cell lines (Huang et al., 2019). The effects of this phosphorylation, along with the existence of other phosphorylatable LIR motifs in the HGF receptor (in the proximity of Y1349, Y1356, and Y1365), remain still to be elucidated.

The possibility that phosphorylation of tyrosines in the Φ_1_ position could be a more general regulatory mechanism for the Y-type LIR is intriguing and deserve further investigation in a high-throughput manner.

## Interaction of ATG8 Family Members With Autophagic Membranes

The tight association of ATG8 proteins with organelle membranes is essential for their main biological functions after autophagy induction (Ichimura et al., 2000). ATG8s have been identified in all autophagic membranes, from the early to the late stages of the process. In this section, we will focus on the mechanisms of interaction of ATG8 proteins with the autophagic membranes and their capability to modulate membrane shape and functionality (Abdollahzadeh et al., 2017).

**FIGURE 6:**
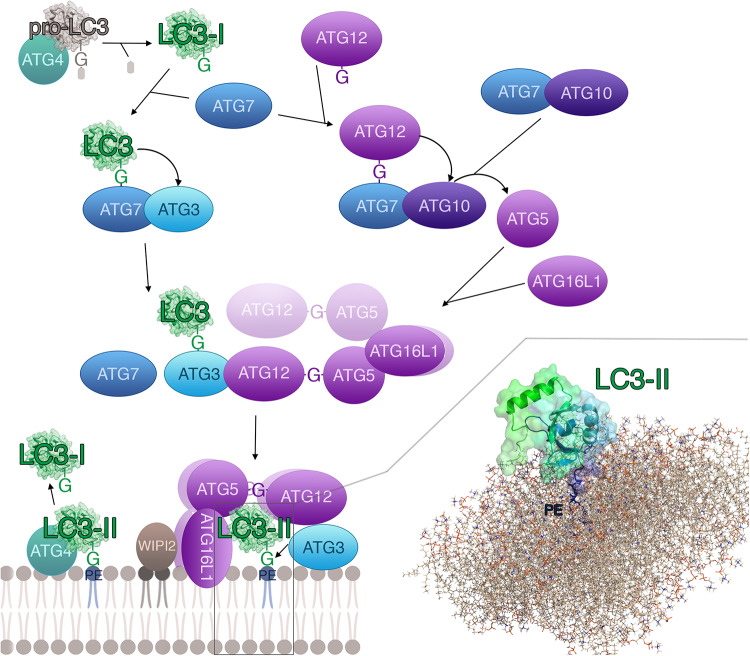
Association of ATG8 proteins to autophagic membranes is important for their biological function. We illustrate the main mechanisms and protein-protein, protein-membrane interactions of the core autophagy machinery members involved in the priming, lipidation and de-lipidation of ATG8 proteins during starvation-induced autophagy. The association of ATG8 to the membranes occurs through its C-terminal conjugation of phosphatidylethanolamine (PE). This process is regulated by proteins in the core autophagy machinery and two ubiquitin-like conjugation systems. In the bottom panel a model of LC3B-PE inside the membrane is shown. The structure of LC3B [PDB entry: 1V49 (Kouno et al., 2005)] is shown as cartoon and surface, using a color gradient from the N-terminal (green) to the C-terminal (dark blue). The PE is shown as dark blue stick and the membrane as light brown sticks.

The association of ATG8 proteins with the lipid membranes occurs through lipid conjugation (Ichimura et al., 2000). The conjugation reaction consists in the covalent and reversible linkage of phosphatidylethanolamine (PE) to the C-terminus of ATG8 proteins, and it is carried out by the proteins composing the two ubiquitin-like conjugation systems (Ichimura et al., 2000; Figure 6). ATG8 proteins are expressed in the cytosol as precursors (pro-ATG8s) characterized by the presence of an extended C-terminal tract with variable length, from one residue in the yeast ATG8 variant to 21 residues in the human LC3C (Ichimura et al., 2000). ATG8 proteins are then processed at the C-terminus by the ATG4 family of cysteine proteases, yielding a cleaved form I with a terminal glycine (ATG8-I). X-ray crystallography and NMR, together with MD simulations, showed that the C-terminus of the ATG8 proteins binds ATG4 in an extended conformation to reach its catalytic site (Satoo et al., 2009). Moreover, ATG4 interacts with ATG8 proteins by a LIR motif in its C-terminal region (Skytte Rasmussen et al., 2017). The E1-like enzyme ATG7 activates the ATG8-I forms, forming an adenylated intermediate and subsequently a thioester bond through a cysteine. This reaction permits the transfer of ATG8-II forms (ATG8-PE) to a cysteine of the E2-like enzyme ATG3, which attaches PE to the C-terminal glycine via an amide linkage (Ichimura et al., 2000; Ohsumi, 2001). ATG8-PE forms are then inserted into the autophagic membranes.

The molecular mechanisms of LC3B association with lipid bilayers have been explored using biochemical assays and coarse-grained (CG) MD simulations (Thukral et al., 2015) with the MARTINI force field (Monticelli et al., 2008). The results from microsecond CG simulations suggest that the insertion of LC3B in membranes occurs through a concerted process, in which the insertions of the two acyl chains happen sequentially. Moreover, LC3B contacts the membrane via a large interface, involving the helix α3, the strands β4 and β5, and a hotspot of basic residues (i.e., K65, R68, and R69). This basic patch is important for the interaction, as attested by the fact that triple mutant variants at these basic sites reduce the formation of LC3B puncta, which are a marker of the number of autophagosomes formed in the cells (Thukral et al., 2015).

Despite membrane association is essential for the functions of ATG8 proteins, experiments with nanodiscs and solution NMR spectroscopy revealed that GABARAP lipidation and insertion into the membrane do not affect its 3D structure and the binding with LIR peptides. In fact, the LIR binding interface on GABARAP is solvent-exposed and not sterically hindered upon the insertion of the protein into the membrane. These regions are located on the side of GABARAP, which is opposite both to the lipidated C-terminal region and to the anchoring site for the nanodisc membrane (Ma et al., 2010; Figure 6). After the insertion of GABARAP into the membrane, only local variations occur in the residues more proximal to the membrane.

An efficient conjugation reaction requires the presence of the ternary ATG12-ATG5-ATG16 complex, that acts as an E3-like enzyme to support the ATG3 function (Hanada et al., 2007; Fujita et al., 2008; Sakoh-Nakatogawa et al., 2013). Phosphatidylinositol 3-phosphate-binding proteins recruit the ATG12-ATG5-ATG16 complex at the level of the surface of the outer membrane of the phagophore, contributing in defining the localization of the lipidation of ATG8 proteins (Dooley et al., 2014). Moreover, at the convex outer face of the phagophore, lipidated ATG8 proteins recruit the ATG12-ATG5-ATG16 complex by interacting with ATG12, forming a continuous, flat, meshwork-like scaffold over the membranes (Kaufmann et al., 2014). On the other hand, cargo receptor proteins engage the ATG12-ATG5-ATG16 complex and promote the formation of lipidated ATG8 proteins on the concave inner surface of the phagophore (Xie et al., 2008). Therefore, ATG8 proteins, through multiple binding sites, promote a complex protein-protein and protein-membrane interaction network on the two surfaces of the phagophore, contributing to its structural and functional asymmetry (Kaufmann et al., 2014). These networks of interactions might stabilize the shape and curvature of the phagophore, contributing to its extension and maturation. Cells can modulate these networks, e.g., through changes in the localization and availability of ATG8 proteins, regulating the size of the autophagosome and the selectivity of the process (Xie et al., 2008; Knorr et al., 2014; Abdollahzadeh et al., 2017). PE-conjugated ATG8 proteins are membrane curvature-sensing proteins, and their activity depends on the curvature of the membrane. Moreover, ATG8 proteins have the propensity to accumulate at the level of highly curved regions of the membranes (Knorr et al., 2014; Nguyen et al., 2017). Changes of shape in artificial vesicles have been associated with the conjugation of ATG8 proteins, suggesting a role in the curvature generation (Knorr et al., 2014). A recent study pointed out that ATG3 identifies the membrane curvature *in vitro* and determines the sensitivity of the lipidated forms of ATG8 to the curvature (Nguyen et al., 2017). This activity might be mediated by the insertion of an N-terminal helix of ATG3 in the packing defects of the lipid bilayer, which are present only in the highly curved regions. *In vivo*, the authors showed that this mechanism has effect on the activity of ATG3 and does not influence its localization (Nguyen et al., 2017). These studies overall support the notion that the ATG8 proteins play a role in modulating some properties of the phagophore membrane such as asymmetry and curvature.

Lipid compositions of liposomes also affect the capability of ATG8 proteins to mediate the fusion of vesicles *in vitro*. This is especially the case of enrichment in lipids that induce negative spontaneous curvature like cardiolipin, diacylglycerol and PE (Landajuela et al., 2016; Abdollahzadeh et al., 2017). Furthermore, ATG8 proteins have intrinsic and evolutionary conserved tethering and fusogenic activities toward membranes. Experiments with an *in vitro* conjugation system from purified proteins show that the yeast ATG8-PE variant mediates tethering of liposomes in large aggregates and hemifusion (i.e., the fusion of the outer leaflet only) of liposomal membranes (Nakatogawa et al., 2007). Experiments with a liposome-based cell-free system demonstrate that lipidated ATG8s, as LC3B and GABARAPL2, promote membrane tethering and fusion (Weidberg et al., 2011). The membrane-modulating activities of ATG8 proteins depend on their conjugation with PE and they are regulated by the ATG4 protein family. These functions are involved in the biogenesis and expansion of the autophagosome (Nakatogawa et al., 2007; Weidberg et al., 2011). Protein-protein interactions between ATG8-PE molecules conjugated with different membranes can mediate liposome tethering. Several studies confirmed the biological activity of ATG8 proteins in membrane tethering and suggested that it is independent of the density of lipidated proteins at the adhesion zone in the membrane (Knorr et al., 2014).

The fusion activity is mostly mediated by the N-terminal tract of ATG8 proteins that contains the helix α1, a region essential and sufficient to induce membrane fusion. LC3B and GABARAPL2 have several positively charged and hydrophobic residues in their N-terminal regions, respectively. These regions might promote membrane tethering and fusion during autophagy, as in the biogenesis of the autophagosomes (Weidberg et al., 2011). The N-terminal regions of the PE-conjugated LC3B and GABARAPL2 in a membrane might undergo conformational changes and be projected toward the facing membrane, interacting with it during the fusion process. Due to the presence of residues with different chemical properties, it is possible that the N-terminal residues of GABARAPL2 could project inside the facing membrane while the N-terminal region of LC3B could interact with the charged head groups of the lipids. These interactions could locally disturb the lipid packing of the membrane and thus induce higher curvature. This mechanism, together with the tethering function, brings the membranes closer, and reduces the energy barrier for the fusion to happen (Martens and McMahon, 2008). Among ATG8 proteins, GABARAP subfamily members have the strongest tethering and fusogenic activities, efficiently mediating the clustering of liposomes and full fusion of both membrane leaflets (Landajuela et al., 2016). The ability to tether liposomes and induce fusion of vesicles is lower for the LC3 subfamily, but they are involved in modulating vesicle shape (Landajuela et al., 2016).

The LC3 subfamily is involved in promoting the extension of precursor membranes. GABARAP subfamily members are, instead, mostly involved in the late stages of autophagy (Nguyen et al., 2016; Vaites et al., 2018). Indeed, the loss of members of the LC3 subfamily causes the formation of autophagosomes of smaller size. In contrast, the loss of proteins of the GABARAP subfamily results in larger autophagosomes and an altered fusion with the lysosomes. These effects could be related either to the loss of interactions with other binding partners or to the lack of activity of the GABARAP proteins in supporting autophagosome-lysosome fusion or closure. In this context, we foresee that future investigations will permit to clarify the contribution, interactions, and specific functions of ATG8 family members in membranes in the spatiotemporal evolution of autophagy. It is indeed crucial to decipher in detail how the membrane association and the diverse functions of ATG8s are regulated and coordinated to increase the knowledge of the molecular mechanisms of autophagy and how they are altered in diseases and cancer.

## Non-LIR Mediated Interaction of ATG8 Proteins With Biological Partners

A full understanding of the binding mechanisms of ATG8 proteins is also complicated by the existence of LIR-independent interactions. In 2010, a pioneer system biology study identified a large fraction of proteins that bind LC3 and GABARAP independently of their LIR docking site (LDS) (Behrends et al., 2010). Despite this study indicated the presence of a novel binding mechanism, in the last decade, a few examples of proteins, which can interact with ATG8 family members in a LIR-independent fashion, have been characterized. A remarkable example is LGG-1 (ATG8 homolog in *C. elegans*), which can interact with the SQSTM1 homolog SQST-1 through a LIR-independent mechanism (Lin et al., 2013). LGG-1 binds two different regions of SQST-1, recapitulating a common binding feature of SLiMs. In a human cellular model, the IAP family member BRUCE promotes autophagosome–lysosome fusion through its interaction with GABARAP, GABARAPL1 and Syntaxin-17, which is a key regulator of the fusion process. BRUCE binds a GABARAP mutant with a mutated LDS, suggesting an alternative mode of interaction (Ebner et al., 2018). In the same context, a recent study illustrates that IRGM promotes the translocation of Syntaxin-17 to the autophagosomes through direct interactions with ATG8 proteins (Kumar et al., 2018). Mutations in the predicted LIR motifs of IRGM or mutations in the ATG8 LDSs do not impair the binding between IRGM and ATG8 proteins, indicating, also in this case, the presence of alternative mechanisms of recognition (Kumar et al., 2018).

**FIGURE 7:**
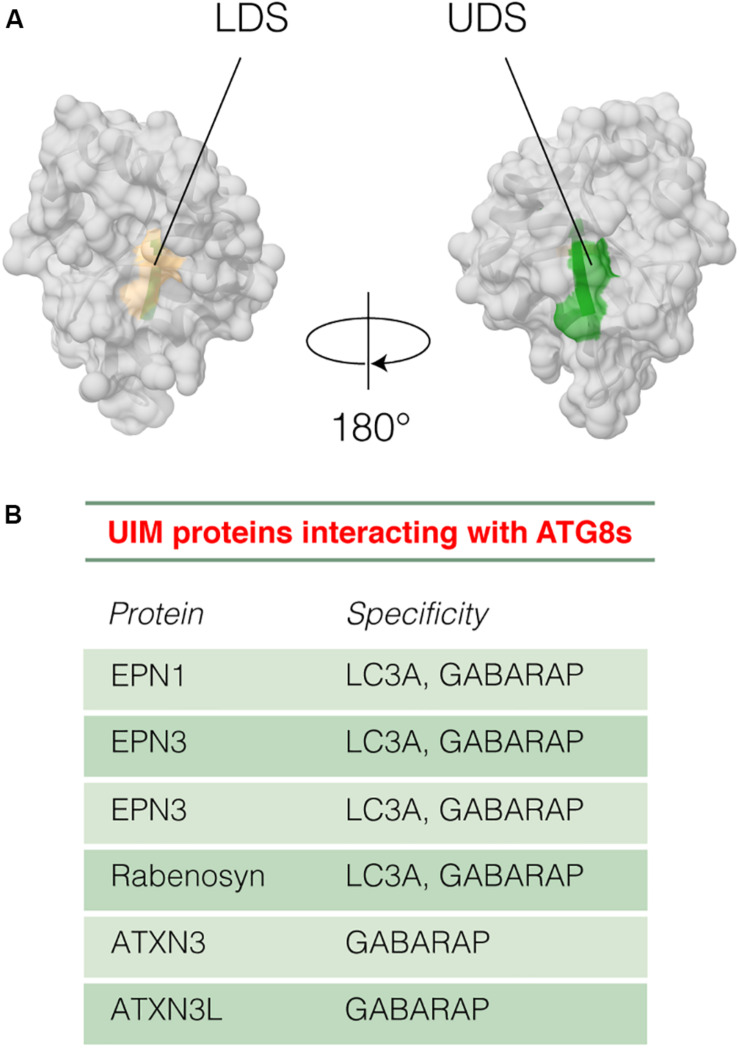
Interaction between UIM motifs and ATG8 proteins. **(A)** The figure illustrates the location on the 3D structure of the yeast ATG8 protein of the LIR Docking Site (LDS, tan) and the UIM Docking Site (USD, green) using the PDB entry 3VXW (Kondo-Okamoto et al., 2012). **(B)** The table shows the UIM-containing proteins that bind LC3A or GABARAP with respective specificities.

While the studies mentioned above did not investigate the interaction at the structural level, a novel interaction interface in the ATG8 structure, which is distant from the conventional LDS, has been identified in plants, human and yeast (Marshall et al., 2019). As a starting point, in 2015, the protein RPN10 was demonstrated to act as a selective autophagy ubiquitin receptor to target inactive 26S proteasomes to autophagosomes in *Arabidopsis* (Marshall et al., 2015). In this work, the authors demonstrated that RPN10 binds to ATG8 through a specific Ubiquitin-Interacting Motif (UIM) (Figure 7). The same team demonstrate, in another work, the presence of a UIM docking site (UDS) of ATG8 characterized by a conserved phenylalanine surrounded by hydrophobic residues, through yeast two-hybrid assays, high-throughput saturating mutagenesis, and pull down experiments (Marshall et al., 2019). The UDS is located on the opposite side of the ATG8 protein with respect to the LDS, and its interaction with UIM-containing proteins is conserved in yeast and human. Through yeast two hybrid screenings, the authors tested the interaction of 28 human UIM-containing proteins with LC3A and GABARAP. The screening allowed to identify six UIM-containing interactors, of which epsins (i.e., EPN1, EPN2, and EPN3) and rabenosyn interact with both LC3A and GABARAP, whereas Ataxin-3 and Ataxin-3L interact specifically with GABARAP.

The aforementioned studies describe LIR-independent binding mechanisms, focusing on proteins that lack LIR motifs or in which LIR mutations do not impact the binding affinity. However, it is possible to speculate that in some cases there may be a co-existence of canonical LIRs together with alternative ATG8-interacting motifs. These additional sequences could be important for the overall binding affinity and could be the target of post-translational modifications, contributing to create an intricate regulatory network. Moreover, the discovery of LIR-independent interactions adds another level of complexity to ATG8 specificity and differences among the different members of the family. In the future, studies with structural biology approaches will be necessary to extend our knowledge on the mechanisms of regulation of the LIR-independent ATG8s interaction and the co-existence of LIR-dependent and independent binding modes.

## Prediction of LIR Motifs

Thanks to the continuous effort to experimentally characterize functional LIR motifs in autophagy-related proteins, about 80 LIR-containing interactors of the ATG8 family have been identified so far. This task requires the employment of time- and resource-consuming techniques ranging from cell and molecular biology assays to biochemical, spectroscopic and crystallographic techniques, not always suitable for high-throughput scans of potential LIR-mediated interactions. In order to meet the growing need for tools allowing fast and accurate identification of LIRs in the scenario of an ever-increasing number of eukaryotic proteomes available, several bioinformatics tools have therefore been developed over the last 15 years (Mohrlüder et al., 2007; Kalvari et al., 2014; Jacomin et al., 2016; Xie et al., 2016).

The first attempt, dating back to 2007, was the construction of a Position-Specific Scoring Matrix (PSSM), whose weights had been trained against data from a phage display screening of a randomized peptide library. This work resulted in the successful identification of the LIR motif of calreticulin and its interaction with GABARAP when used against the whole SwissProt database (Mohrlüder et al., 2007). Unfortunately, the actual PSSM has never been made available. This fact, combined with the increasing number of experimental data collected in the following years, prompted the development of other approaches such as iLIR (Kalvari et al., 2014; Jacomin et al., 2016) and hfAIM (Xie et al., 2016). Both iLIR and hfAIM are rooted in the definition of the LIR motif as a regular expression pattern. The idea was first introduced by Alemu et al. (2012) and subsequently expanded to include the contributions of residues N- and C-terminal to the core 4-residues sequence, as soon as the importance of the flanking regions became clear (Jacomin et al., 2016). The LIR motif is also identified by a regular expression pattern in the Eukaryotic Linear Motif database (Puntervoll et al., 2003), and the entry has been extensively revised in the last database update (Kumar et al., 2019). On the other hand, the iLIR “extended” LIR motif, named xLIR (Kalvari et al., 2014) matched by design the 27 experimentally verified LIRs available at that time. In contrast, hfAIM includes five different patterns to describe possible LIR motifs also designed to match the available experimental data. Although the performance of both methods on the testing datasets was far from perfect, the authors of iLIR demonstrated that the performances of their method could be sensibly improved by adding (after the pattern-matching step) a PSSM trained against the experimentally validated LIRs, and defining an empirical score threshold discriminating between putative LIRs and non-significant hits. Then, they also improved iLIR performances by coupling the PSSM with the ANCHOR predictor for disordered sequences (Mészáros et al., 2009; Kalvari et al., 2014), incorporating the observation that autophagy-related proteins are often abundant in intrinsically disordered regions (Mei et al., 2014).

Nevertheless, given the rapid expansion of the dataset of experimentally validated LIRs, the deeper understanding of the role of residues flanking the core residues and the advances in capturing the sequence and structural determinants of the specificity toward the different ATG8 proteins (Rogov et al., 2017a; Atkinson et al., 2019; Jatana et al., 2019; Wirth et al., 2019), it may be possible to devise different representations of the LIR motif using more sophisticated machine learning techniques, as already done for the discovery of other SLiMs (Davey et al., 2009; Mooney et al., 2012; Palopoli et al., 2015; Krystkowiak and Davey, 2017) and integrating data from orthogonal sources of information (i.e., available structures, co-evolving sequences, functional annotations, etc.), to find new fundamental players in the diverse, complex and finely tuned mechanisms of autophagy.

## Concluding Remarks

The ATG8 family of proteins is central in different steps of selective autophagy, thanks to a large network of protein-protein interactions and association with biological membranes. Different ATG8 family members can also play diverse roles in a context-dependent manner, adding complexity to complexity. We provide a comprehensive review and curation of the structural and computational studies on the human ATG8 proteins and identify outstanding questions in the field. Our contribution can serve as a roadmap for future studies in the field. The first MD simulations in this field appeared in the last 5 years and already attested their potential to address questions related to specificity, impact of mutations and related dynamics and function of this class of proteins. As a field in its infancy, modeling and simulations of these proteins still require methodological efforts with the aim of identifying the physical models that better suit this group of proteins and their post-translational modifications. These aspects would benefit from the availability of different NMR measurements and chemical shift assignments and other biophysical experiments to use for simulation validation and assessment. The possibility of integrating experimental data directly in the simulation protocols has also been poorly explored for these proteins. Molecular mechanisms, such as modulation or changes induced by post-translational modifications, interactions with lipids and assessment of proline-based conformational switches emerge, for example, as interesting directions for future computational research.

## Author Contributions

VS contributed to the writing of sections “The Interaction Between Human ATG8 Proteins and Their Biological Partners Through Short Linear Motifs, i.e., the LC3 Interacting Regions (LIRs),” “Specificity of Different ATG8 Family Members in LIR Recognition,” “Post-translational Modulation of the LIR-ATG8s Interaction,” and Figure 5, Table 1, and Supplementary Table S1. MK contributed to the writing of sections “Introduction,” “ATG8 Family Members in Human and Their Conservation,” “Structure of ATG8 Family Members,” and “Specificity of Different ATG8 Family Members in LIR Recognition,” and Figure 2. EM contributed to the writing of section “Non-LIR Mediated Interaction of ATG8 Proteins With Biological Partners” and Figure 7. ML contributed to the writing of sections “Structural Dynamics of ATG8 Family Members” and “Interaction of ATG8 Family Members With Autophagic Membranes,” and Figures 1, 3, 4 and 6. MT contributed to the writing of sections “The Interaction Between Human ATG8 Proteins and Their Biological Partners Through Short Linear Motifs, i.e., the LC3 Interacting Regions (LIRs),” “Specificity of Different ATG8 Family Members in LIR Recognition,” and “Post-translational Modulation of the LIR-ATG8s Interaction,” and Figure 2. EP contributed to all the sections and the final conclusions. All the authors revised the final version of the manuscript and contributed with figures and tables.

## Conflict of Interest

The authors declare that the research was conducted in the absence of any commercial or financial relationships that could be construed as a potential conflict of interest.

## Footnotes

1 https://github.com/ELELAB/PCM1_LIR

## Supplementary Material

TABLE S1 Known experimentally-determined structures of either member of the ATG8 family in free state or in complex with a biological partner interacting via LIR motif. We report the PDB entry for each structure, along with information on the ATG8 protein, the interactor (if present), the experimental technique used for structure determination, the atomic resolution of the structure, the PMID of the associated publication and additional notes. The resolution was predicted with ResProx for structures resolved employing solution NMR.

DATA SHEET S1 Full list of abbreviations used in the text.

## Abbreviations

The full list of abbreviations is in the Supplementary File Data Sheet S1.
