# Supplementary material for: Structure and Dynamics in the ATG8 Family From Experimental to Computational Techniques
Source: Front Cell Dev Biol. 2020 Jun 10;8:420. doi: 10.3389/fcell.2020.00420 (PMC7297954; doi:10.3389/fcell.2020.00420)
Supplement: TABLE S1 — Known experimentally-determined structures of either member of the ATG8 family in free state or in complex with a biological partner interacting via LIR motif. We report the PDB entry for each structure, along with information on the ATG8 protein, the interactor (if present), the experimental technique used for structure determination, the atomic resolution of the structure, the PMID of the associated publication and additional notes. The resolution was predicted with ResProx for structures resolved employing solution NMR. [file Table_1.DOCX]

| PDB ID | ATG8 protein | Partner | Technique | Resolution (Å) | Associated publication (PMID) | Notes |
| --- | --- | --- | --- | --- | --- | --- |
| 3ECI | LC3A | /// | X-ray | 2.65 | /// | /// |
| 3WAL | LC3A | /// | X-ray | 2 | 24290141 | /// |
| 3WAN | LC3A | /// | X-ray | 1.77 | 24290141 | /// |
| 1V49 | LC3B | /// | Solution NMR | 2.643 | 15857831 | /// |
| 3VTU | LC3B | /// | X-ray | 1.6 | 23805866 | /// |
| 2NCN | LC3C | /// | Solution NMR | 2.073 | /// | /// |
| 3WAM | LC3C | /// | X-ray | 1.75 | 24290141 | /// |
| 1GNU | GABARAP | /// | X-ray | 1.75 | 11729197 | /// |
| 1KLV | GABARAP | /// | Solution NMR | 3.044 | 11885988 | /// |
| 1KJT | GABARAP | /// | X-ray | 2 | 11818336 | /// |
| 1KM7 | GABARAP | /// | Solution NMR | 2.626 | 11885988 | /// |
| 1KOT | GABARAP | /// | Solution NMR | 2.819 | 11875056 | /// |
| 2R2Q | GABARAPL1 | /// | X-ray | 1.65 | /// | /// |
| 1EQ6 | GABARAPL2 | /// | X-ray | 1.9 | 10860733 | /// |
| 4CO7 | GABARAPL3 | /// | X-ray | 2 | 26284781 | /// |
| 3WIM | GABARAP | Alfy | X-ray | 2.6 | 24668264 | /// |
| 5YIR | GABARAP | Ankyrin-2 | X-ray | 2.75 | 29867141 | GABARAP from *M. musculus* |
| 5YIS | LC3B | Ankyrin-2 | X-ray | 2.201 | 29867141 | LC3B from *M. musculus* |
| 5YIP | GABARAPL1 | Ankyrin-3 | X-ray | 1.85 | 29867141 | GABARAPL1 from *M. musculus*, Ankyrin-3 from *R. norvegicus* |
| 5YIQ | LC3B | Ankyrin-3 | X-ray | 2.6 | 29867141 | LC3B from *M. musculus*, Ankyrin-3 from *R. norvegicus* |
| 6A9X | GABARAP | Ankyrin-3 | X-ray | 2.202 | 30504823 | GABARAP from *M. musculus*, Ankyrin-3 from *R. norvegicus* |
| 5LXH | GABARAPL1 | ATG4B | X-ray | 1.58 | 28287329 | /// |
| 5LXI | GABARAPL1 | ATG4B | X-ray | 1.44 | 28287329 | /// |
| 3WAN | LC3A | ATG13 | X-ray | 1.77 | 24290141 | chimeric protein |
| 3WAP | LC3C | ATG13 | X-ray | 3.1 | 24290141 | chimeric protein |
| 3WAO | LC3B | ATG13 | X-ray | 2.6 | 24290141 | chimeric protein |
| 6HYN | GABARAP | ATG13 | X-ray | 1.14 | 31053714 | chimeric protein |
| 6HOL | GABARAPL1 | ATG14 | X-ray | 1.4 | 30767700 | /// |
| 6HOI | GABARAPL1 | Beclin-1 | X-ray | 1.14 | 30767700 | /// |
| 6HOJ | GABARAP | Beclin-1 | X-ray | 1.51 | 30767700 | chimeric protein |
| 6HOK | GABARAP | Beclin-1 | X-ray | 1.61 | 30767700 | chimeric protein, S96E in Beclin-1 |
| 3DOW | GABARAP | Calreticulin | X-ray | 2.3 | 19154346 | /// |
| 4ZDV | LC3A | RETREG1 | X-ray | 1.8 | 26040720 | chimeric protein |
| 2N9X | LC3B | FUNDC1 | Solution NMR | 1.599 | 27653272 | /// |
| 5GMV | LC3B | FUNDC1 | X-ray | 2.25 | 27757847 | /// |
| 5CX3 | LC3A | FYCO1 | X-ray | 2.3 | 27246247 | /// |
| 5D94 | LC3B | FYCO1 | X-ray | 1.53 | 26468287 | /// |
| 4XC2 | GABARAP | KBTBD6 | X-ray | 1.9 | 25684205 | /// |
| 2L8J | GABARAPL1 | NBR1 | Solution NMR | 2.224 | 21620860 | /// |
| 3VVW | LC3C | CALCOCO2 | X-ray | 2.5 | 23022382 | /// |
| 5V4K | LC3B | NEDD4 | X-ray | 2.099 | 28470758 | chimeric protein |
| 4WAA | LC3B | BNIP3L | X-ray | 2.35 | 28442745 | chimeric protein |
| 2LUE | LC3B | Optineurin | Solution NMR | 2.037 | 23805866 | pS170, pS171, pS173, pS174, pS177 in Optineurin |
| 3VTV | LC3B | Optineurin | X-ray | 1.7 | 23805866 | chimeric protein |
| 3VTW | LC3B | Optineurin | X-ray | 2.52 | 23805866 | chimeric protein, S177E in Optineurin |
| 2K6Q | LC3B | SQSTM1 | Solution NMR | 2.968 | /// | LC3B and Sqstm1 from *R. norvegicus* |
| 2ZJD | LC3B | SQSTM1 | X-ray | 1.56 | 18524774 | Sqstm1 from *M. musculus* |
| 6HYM | GABARAP | PCM1 | X-ray | 1.86 | 31053714 | chimeric protein |
| 6HYL | GABARAP | PCM1 | X-ray | 1.559 | 31053714 | chimeric protein |
| 6HOG | GABARAP | PI3K type 3 | X-ray | 1.26 | 30767700 | chimeric protein |
| 6HOH | GABARAP | PI3K type 3 | X-ray | 2.25 | 30767700 | chimeric protein, S249E in PIK3K type 3 |
| 3X0W | LC3B | PLEKHM1 | X-ray | 2.71 | 25498145 | chimeric protein |
| 5DPS | GABARAP | PLEKHM1 | X-ray | 2 | 28655748 | chimeric protein |
| 5DPT | GABARAPL1 | PLEKHM1 | X-ray | 2.9 | 28655748 | chimeric protein |
| 5DPR | LC3A | PLEKHM1 | X-ray | 2.5 | 28655748 | chimeric protein |
| 5DPW | LC3C | PLEKHM1 | X-ray | 2.185 | 28655748 | /// |
| 5DCN | LC3B | TECPR2 | X-ray | 2 | 26431026 | chimeric protein |
| 5W9A | LC3B | TRIM5 | X-ray | 2.74 | 30282803 | /// |
| 6HB9 | GABARAP | UBA5 | X-ray | 1.3 | 30990354 | chimeric protein |
| 6H8C | GABARAPL2 | UBA5 | Solution NMR | 2.06 | 30990354 | /// |
| 6HYO | GABARAP | ULK1 | X-ray | 1.07 | 31053714 | chimeric protein |
